# Supplementary material for: Bias and negative values of COVID-19 vaccine effectiveness estimates from a test-negative design without controlling for prior SARS-CoV-2 infection
Source: Nat Commun. 2024 Nov 20;15:10062. doi: 10.1038/s41467-024-54404-w (PMC11579392; doi:10.1038/s41467-024-54404-w)
Supplement: Supplementary file 1 — Supplementary Information [file 41467_2024_54404_MOESM1_ESM.pdf]

Supplementary materials for 'Bias and negative values of vaccine effectiveness estimates from a test-negative design without controlling for prior infection'

Ryan E. Wiegand, Bruce Fireman, Morgan Najdowski, Mark W. Tenforde, Ruth Link-Gelles, Jill M. Ferdinands

Contents

Supplemental Results 2
Supplemental Tables 2
Supplemental Figures 5
VE against symptomatic infection 8
VE against severe disease 21
Simulation methods 34
Part 1: historical period 34
Probability of cases 34
Probability of vaccination 36
Protection 39
Generation of infection and vaccination 44
Example plot of protection 45
Part 2: analytic period 47
Probability of cases 47
Probability of vaccination 48
Symptoms 49
Test Characteristics 50
Symptomatic infection outcome 51
Severe disease outcome 51
Analytic methods 53
Models 53
Bias 54
Unstable estimates 54
References 56

Supplemental Results

Supplemental Tables

Supplementary Table 1. Median, minimum, and maximum per simulation of protection against infection, protection against severe disease, weeks since vaccination, and weeks since infection across 200 simulated populations for each of the 8 combinations of vaccine protection, infection protection, and hybrid protection definitions.

Vaccine protection defintion (VP)	Infection protection defintion (IP)	Hybrid protection defintion (HP)	Median protection against infection per simulation median (min, max)	Median protection against severe disease per simulation median (min, max)	Median weeks since vaccination per simulation median(min, max)	Median weeks since infection per simulation median(min, max)
VP wanes by 48(24) wks	IP wanes by 96 wks	Additional 30% either	0.46 (0.33, 0.51)	0.94 (0.90, 0.96)	68.0 (68.0, 69.0)	45.0 (41.0, 52.0)
VP wanes by 24(12) wks	IP wanes by 96 wks	Additional 30% either	0.46 (0.31, 0.51)	0.95 (0.89, 0.97)	68.0 (68.0, 69.0)	46.0 (41.0, 53.0)
VP wanes by 48(24) wks	IP wanes by 72 wks	Additional 30% either	0.37 (0.26, 0.44)	0.95 (0.92, 0.97)	68.0 (67.0, 69.0)	42.0 (36.0, 47.0)
VP wanes by 24(12) wks	IP wanes by 72 wks	Additional 30% either	0.36 (0.26, 0.43)	0.96 (0.93, 0.97)	68.0 (68.0, 69.0)	42.0 (37.0, 47.0)
VP wanes by 48(24) wks	IP wanes by 96 wks	Additional 10% VP or 30% IP	0.46 (0.40, 0.50)	0.89 (0.87, 0.91)	68.0 (68.0, 69.0)	45.0 (41.0, 49.0)
VP wanes by 24(12) wks	IP wanes by 96 wks	Additional 10% VP or 30% IP	0.46 (0.35, 0.50)	0.90 (0.87, 0.91)	68.0 (68.0, 69.0)	46.0 (41.0, 51.0)
VP wanes by 48(24) wks	IP wanes by 72 wks	Additional 10% VP or 30% IP	0.36 (0.26, 0.44)	0.90 (0.87, 0.92)	68.0 (67.0, 69.0)	42.0 (36.0, 47.0)
VP wanes by 24(12) wks	IP wanes by 72 wks	Additional 10% VP or 30% IP	0.36 (0.24, 0.43)	0.90 (0.87, 0.92)	68.0 (68.0, 69.0)	42.0 (37.0, 48.0)

Supplementary Table 2. Median, minimum, and maximum per simulation for protection against infection, protection against severe disease, weeks since vaccination, and weeks since infection across 1,600 simulated populations broken down by number of vaccinations.

Number of vaccinations	N	Median protection against infection per simulation median(min, max)	Median protection against severe disease per simulation median(min, max)	Median weeks since vaccination per simulation median(min, max)	N with prior infection per simulation	Median weeks since infection per simulation median(min, max)
0	36454 (35996, 36929)	0.42 (0.24, 0.52)	0.83 (0.79, 0.86)	-	35904 (32348, 36687)	44.0 (35.0, 56.0)
2	24820 (24392, 25285)	0.41 (0.24, 0.52)	0.91 (0.85, 0.99)	104.0 (103.0, 104.0)	24306 (21823, 25179)	44.0 (36.0, 56.0)
3	14011 (13600, 14358)	0.43 (0.27, 0.52)	0.95 (0.88, 0.99)	72.0 (72.0, 73.0)	13649 (12194, 14107)	43.0 (35.0, 51.0)
4	24725 (24348, 25147)	0.40 (0.21, 0.50)	0.99 (0.99, 0.99)	28.0 (28.0, 29.0)	22708 (17249, 24462)	44.0 (38.0, 49.0)

Supplementary Table 3. Median, minimum, and maximum protection against infection, protection against severe disease, weeks since vaccination, and weeks since infection across 1,600 simulated populations broken down by number of number of infections

Number of infections	N	Median protection against infection per simulation median(min, max)	Median protection against severe disease per simulation median(min, max)	N vaccinated	Median weeks since vaccination per simulation median(min, max)	Median weeks since infection per simulation median(min, max)
0	3414 (794, 15379)	0.00 (0.00, 0.00)	0.70 (0.60, 0.73)	2859 (679, 11885)	32.0 (31.0, 33.0)	-
1	27370 (13460, 48763)	0.18 (0.05, 0.29)	0.92 (0.87, 0.98)	20260 (10397, 32447)	61.0 (35.0, 70.0)	67.0 (65.0, 69.0)
2	39506 (28711, 42006)	0.43 (0.35, 0.51)	0.93 (0.90, 0.99)	24788 (16167, 26657)	71.0 (67.0, 78.0)	43.0 (41.0, 46.0)
3	21685 (6267, 32009)	0.61 (0.55, 0.65)	0.93 (0.90, 0.98)	11824 (3159, 18847)	75.0 (71.0, 82.0)	26.0 (23.0, 28.0)
4	5914 (813, 14218)	0.67 (0.64, 0.70)	0.92 (0.89, 0.97)	2918 (391, 7516)	77.0 (72.0, 88.0)	17.0 (16.0, 18.0)
>=5	1292 (67, 5236)	0.69 (0.65, 0.72)	0.89 (0.85, 0.94)	586 (30, 2444)	80.0 (68.0, 100.0)	14.0 (12.0, 16.0)

Supplemental Figures

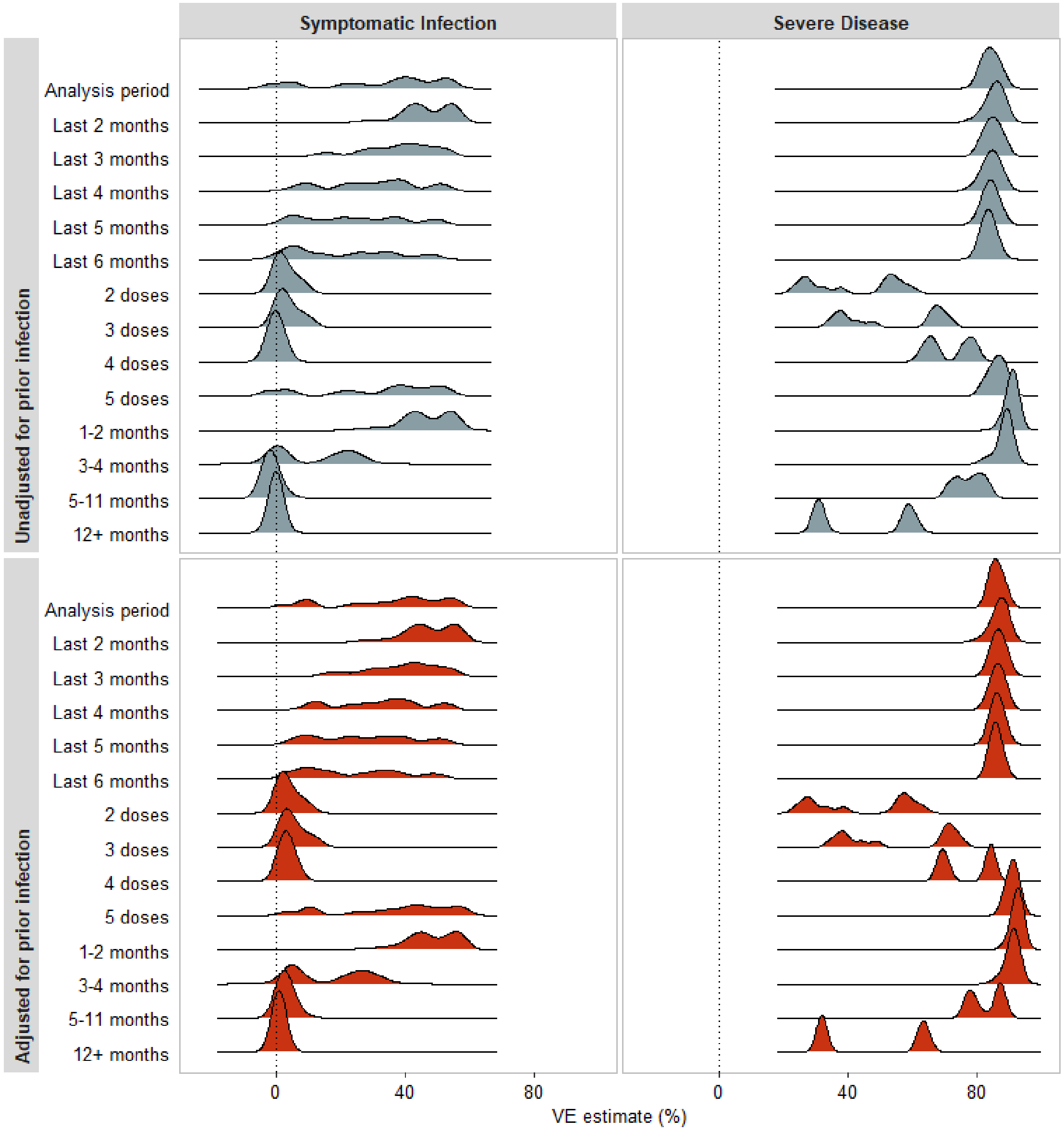

Supplementary Fig. 1: Distribution of simulated VE estimates for VE against symptomatic infection and VE against severe disease. Estimates are for each exposure definition stratifed by estimates unadjusted for prior infection and adjusted for prior infection.

Supplementary Fig. 1 notes: Distributions are comprised of 768 data points, one VE estimate from each parameter set. Unadjusted models are defined in the Analytic Methods section and adjusted models include participants' months since last infection and the number of prior infections in to the unadjusted models.

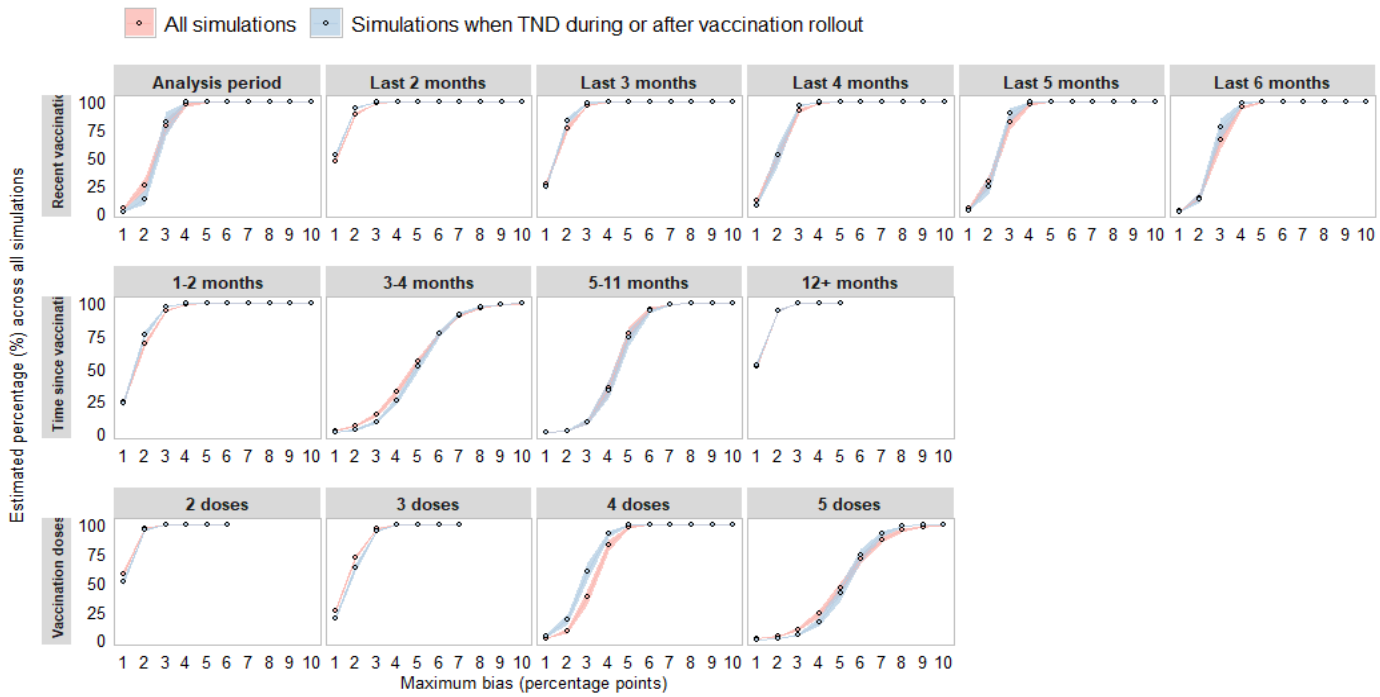

Supplementary Fig. 2: Sensitivity analyses of including or not including simulations with the TND before the vaccination rollout. Plots are the estimated percentage of bias less than or equal to a percentage point threshold for VE against symptomatic infection for multiple exposures.

Supplementary Fig. 2 Caption: Notes: Bias is computed as the difference between VE calculated from the model that does not adjust for prior infection ("unadjusted") and the model adjusted for prior infection ("adjusted"). Bias estimates are generated from a meta-regression of aggregated results from 768 simulation conditions, each of which were summarized from 1,000 simulations. Estimates are presented as the marginal mean estimate (as dots) +/- the 95% confidence interval (represented by bands connecting the maximum percentage point bias thresholds) that are a product of the standard error estimate and normal distribution quantiles.

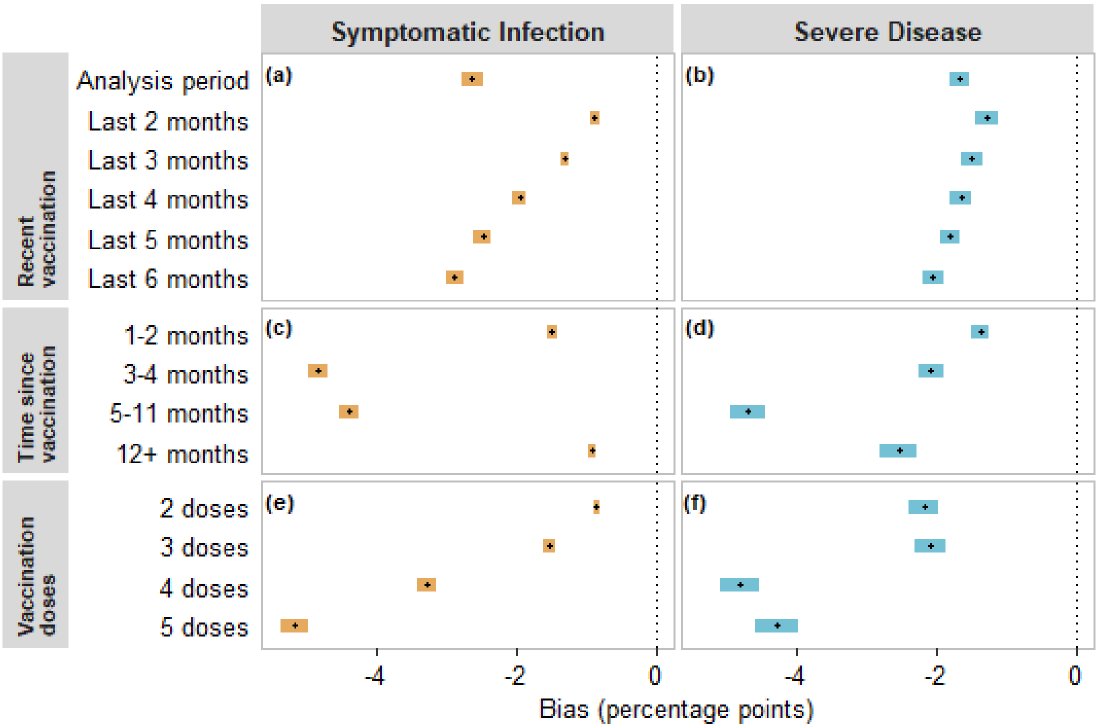

Supplementary Fig. 3: Plot of estimated marginal means of bias of VE against symptomatic infection and VE against severe disease for each exposure.

Supplementary Fig. 3 Notes: VE estimates are generated from a simple meta-regression of 768 simulation conditions each summarized from 1,000 simulations without controlling for simulation parameters. Estimates are presented as the marginal mean (as dots) +/- the 95% confidence interval (represented by bars) that are a product of the standard error and normal distribution quantiles. Panel identifiers are: (a) recent vaccination exposures for VE against symptomatic infection; (b) recent vaccination exposures for VE against severe disease; (c) time since vaccination exposures for VE against symptomatic infection; (d) time since vaccination exposures for VE against severe disease; (e) vaccination dose exposures for VE against symptomatic infection; and (f) vaccination dose exposures for VE against severe disease

VE against symptomatic infection

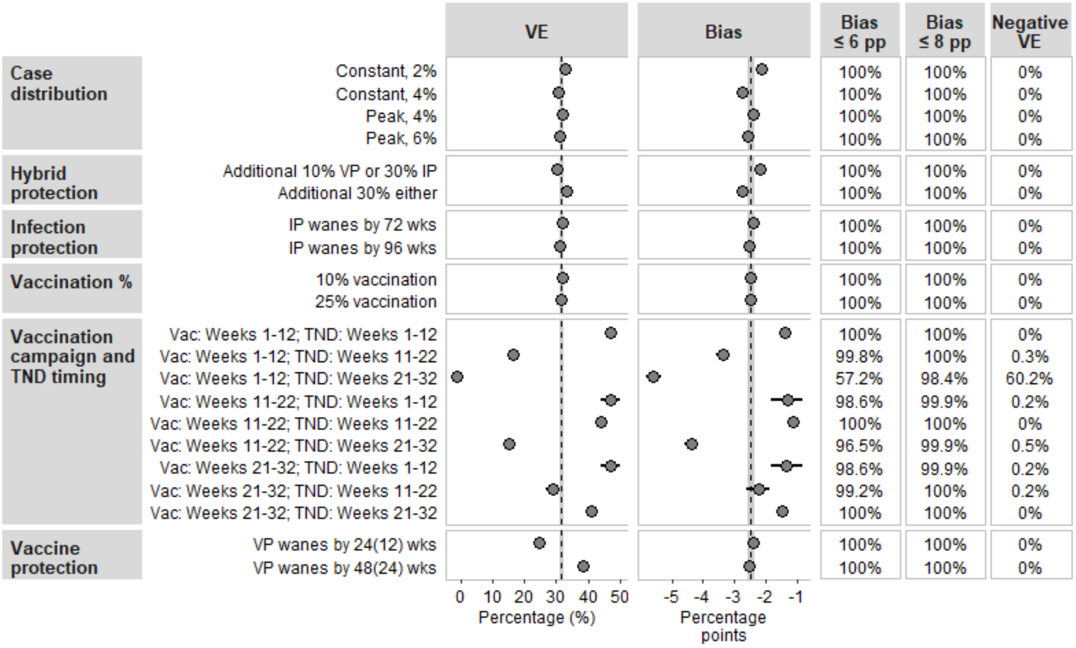

Supplementary Fig. 4: Plot of estimated marginal means of unadjusted VE (not controlling for prior infection) against symptomatic infection, bias compared to adjusted VE (controlling for prior infection) against symptomatic infection, the percentage of simulations with a bias less than or equal to 6 percentage points (pp), 8 pp, and VE estimate less than zero (negative VE) for vaccination at any time during the analytic period.

Supplementary Fig. 4 Notes: Estimates are generated from a meta-regression of aggregated results from 768 simulation conditions (each of which were summarized from 1,000 simulations) after controlling for all other simulation parameters. Bias is computed as the difference between the unadjusted VE estimate compared to the VE estimate adjusted for prior infection. Estimates are presented as the marginal mean (as dots) +/- the 95% confidence interval (represented by solid lines) that are a product of the standard error and normal distribution quantiles. The bars may be narrower than the dot and not visible. The dashed line and shaded region in the VE column represent the overall mean and the 95% confidence interval, respectively, from Fig. 1. In the Bias column, the dashed line and shaded region represent the overall mean and the 95% confidence interval, respectively, from Supplementary Fig. 3.

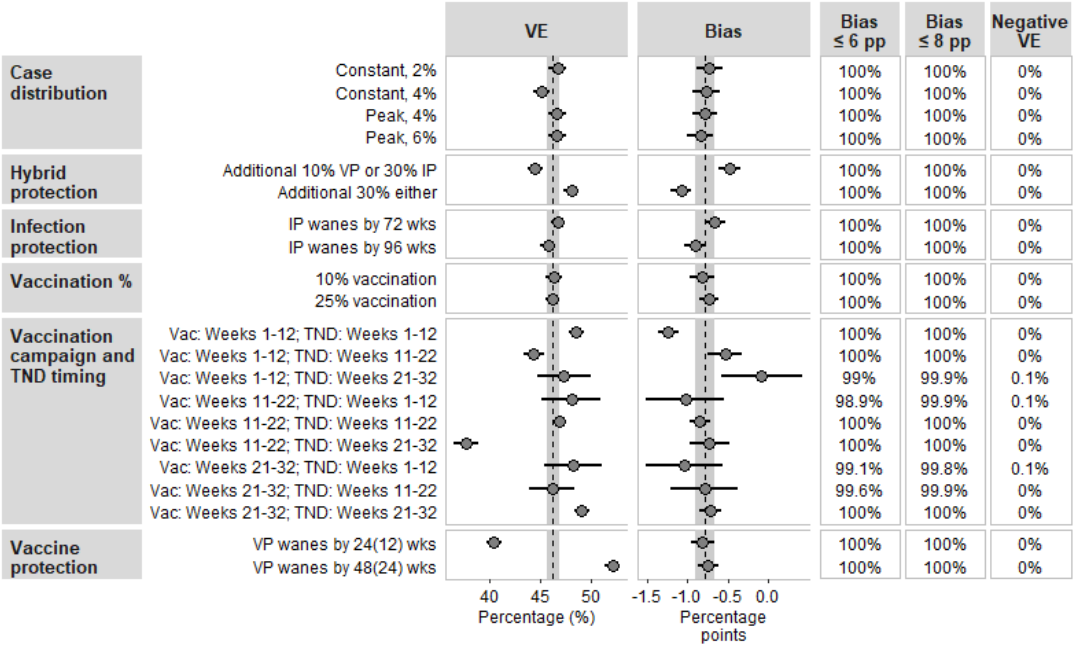

Supplementary Fig. 5: Plot of estimated marginal means of unadjusted VE (not controlling for prior infection) against symptomatic infection, bias compared to adjusted VE (controlling for prior infection) against symptomatic infection, the percentage of simulations with a bias less than or equal to 6 percentage points (pp), 8 pp, and VE estimate less than zero (negative VE) for vaccination in the previous 2 months.

Supplementary Fig. 5 Notes: Estimates are generated from a meta-regression of aggregated results from 768 simulation conditions (each of which were summarized from 1,000 simulations) after controlling for all other simulation parameters. Bias is computed as the difference between the unadjusted VE estimate compared to the VE estimate adjusted for prior infection. Estimates are presented as the marginal mean (as dots) +/- the 95% confidence interval (represented by solid lines) that are a product of the standard error and normal distribution quantiles. The bars may be narrower than the dot and not visible. The dashed line and shaded region in the VE column represent the overall mean and the 95% confidence interval, respectively, from Fig. 1. In the Bias column, the dashed line and shaded region represent the overall mean and the 95% confidence interval, respectively, from Supplementary Fig. 3.

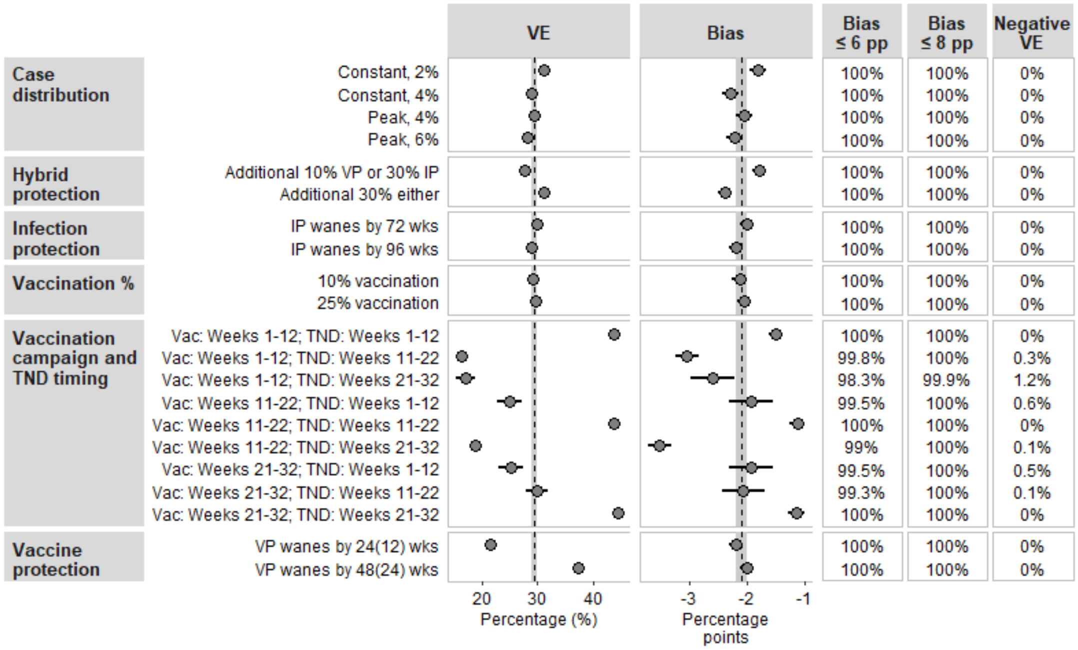

Supplementary Fig. 6: Plot of estimated marginal means of unadjusted VE (not controlling for prior infection) against symptomatic infection, bias compared to adjusted VE (controlling for prior infection) against symptomatic infection, the percentage of simulations with a bias less than or equal to 6 percentage points (pp), 8 pp, and VE estimate less than zero (negative VE) for vaccination in the previous 4 months.

Supplementary Fig. 6 Notes: Estimates are generated from a meta-regression of aggregated results from 768 simulation conditions (each of which were summarized from 1,000 simulations) after controlling for all other simulation parameters. Bias is computed as the difference between the unadjusted VE estimate compared to the VE estimate adjusted for prior infection. Estimates are presented as the marginal mean (as dots) +/- the 95% confidence interval (represented by solid lines) that are a product of the standard error and normal distribution quantiles. The bars may be narrower than the dot and not visible. The dashed line and shaded region in the VE column represent the overall mean and the 95% confidence interval, respectively, from Fig. 1. In the Bias column, the dashed line and shaded region represent the overall mean and the 95% confidence interval, respectively, from Supplementary Fig. 3.

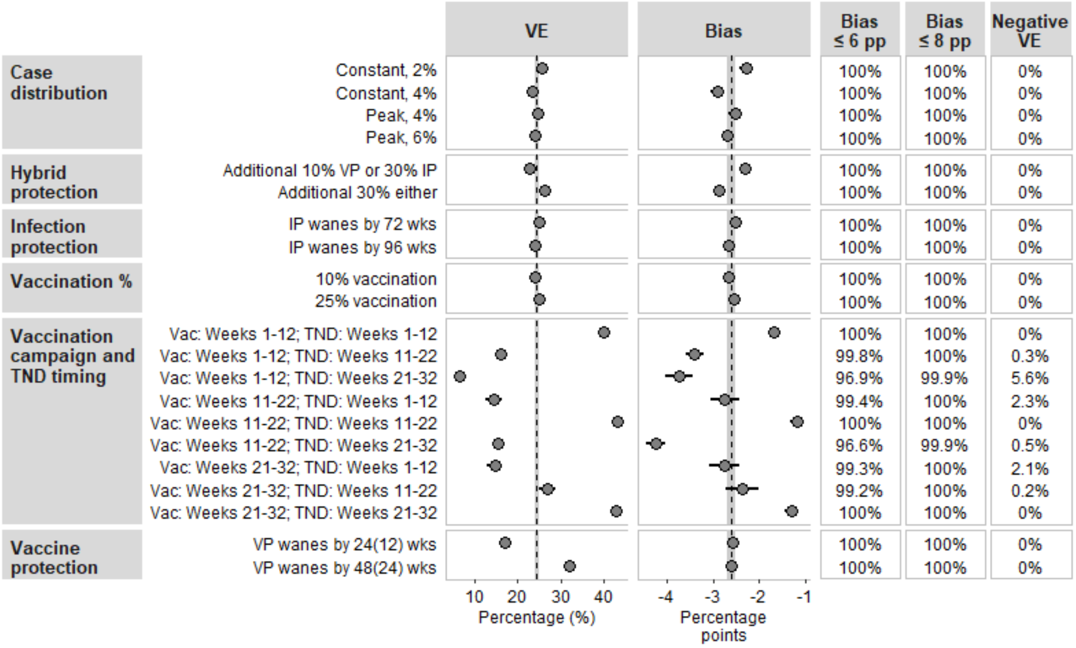

Supplementary Fig. 7: Plot of estimated marginal means of unadjusted VE (not controlling for prior infection) against symptomatic infection, bias compared to adjusted VE (controlling for prior infection) against symptomatic infection, the percentage of simulations with a bias less than or equal to 6 percentage points (pp), 8 pp, and VE estimate less than zero (negative VE) for vaccination in the previous 5 months.

Supplementary Fig. 7 Notes: Estimates are generated from a meta-regression of aggregated results from 768 simulation conditions (each of which were summarized from 1,000 simulations) after controlling for all other simulation parameters. Bias is computed as the difference between the unadjusted VE estimate compared to the VE estimate adjusted for prior infection. Estimates are presented as the marginal mean (as dots) +/- the 95% confidence interval (represented by solid lines) that are a product of the standard error and normal distribution quantiles. The bars may be narrower than the dot and not visible. The dashed line and shaded region in the VE column represent the overall mean and the 95% confidence interval, respectively, from Fig. 1. In the Bias column, the dashed line and shaded region represent the overall mean and the 95% confidence interval, respectively, from Supplementary Fig. 3.

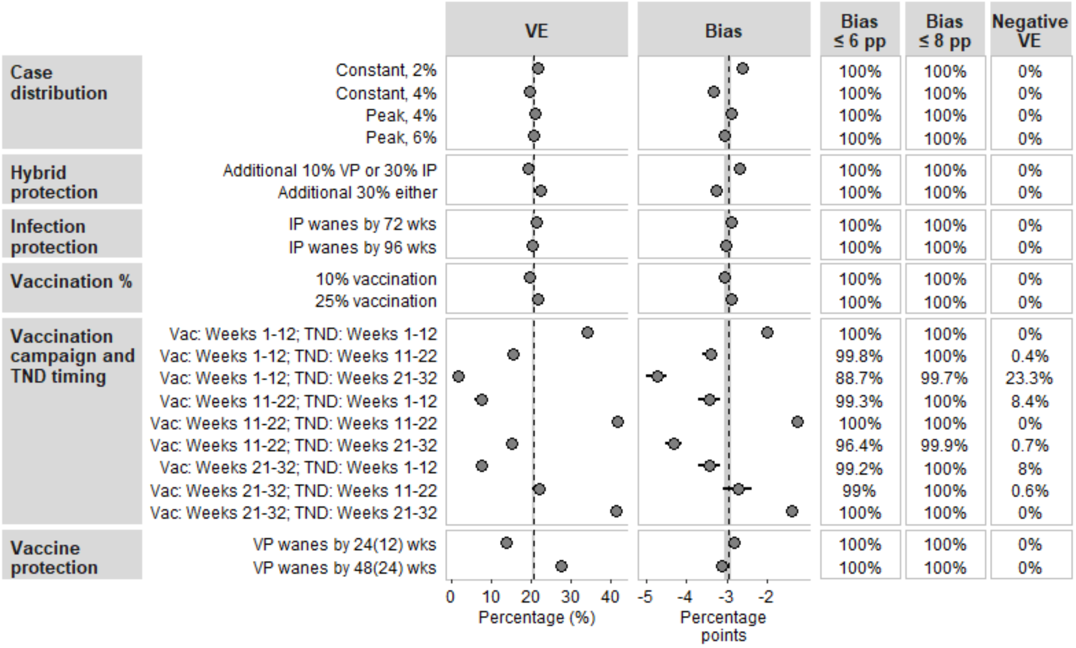

Supplementary Fig. 8: Plot of estimated marginal means of unadjusted VE (not controlling for prior infection) against symptomatic infection, bias compared to adjusted VE (controlling for prior infection) against symptomatic infection, the percentage of simulations with a bias less than or equal to 6 percentage points (pp), 8 pp, and VE estimate less than zero (negative VE) for vaccination in the previous 6 months.

Supplementary Fig. 8 Notes: Estimates are generated from a meta-regression of aggregated results from 768 simulation conditions (each of which were summarized from 1,000 simulations) after controlling for all other simulation parameters. Bias is computed as the difference between the unadjusted VE estimate compared to the VE estimate adjusted for prior infection. Estimates are presented as the marginal mean (as dots) +/- the 95% confidence interval (represented by solid lines) that are a product of the standard error and normal distribution quantiles. The bars may be narrower than the dot and not visible. The dashed line and shaded region in the VE column represent the overall mean and the 95% confidence interval, respectively, from Fig. 1. In the Bias column, the dashed line and shaded region represent the overall mean and the 95% confidence interval, respectively, from Supplementary Fig. 3.

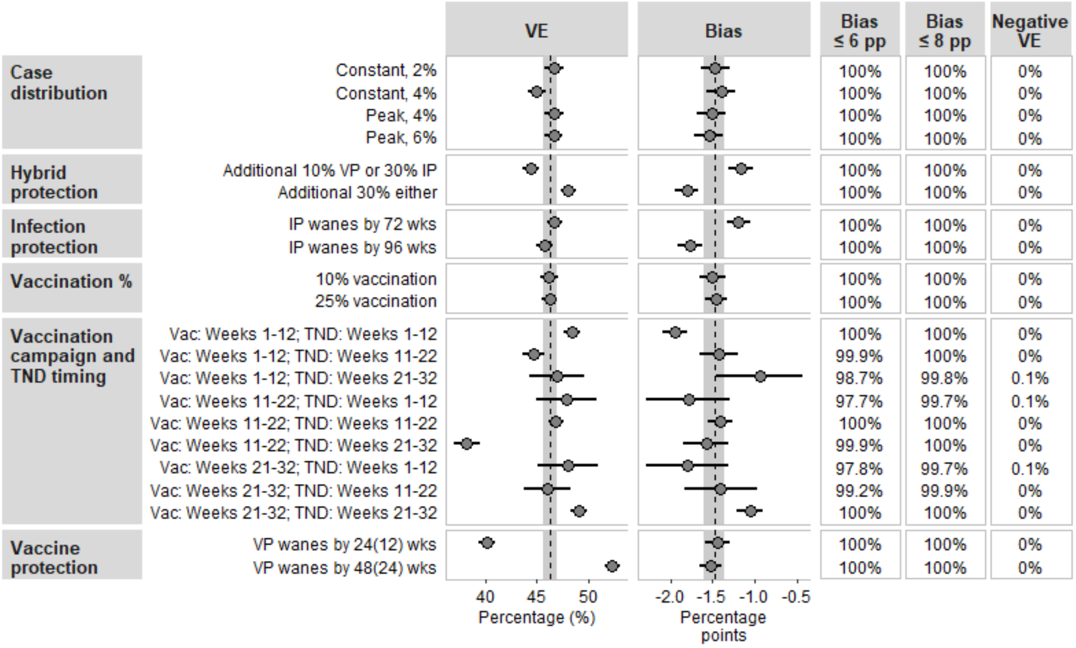

Supplementary Fig. 9: Plot of estimated marginal means of unadjusted VE (not controlling for prior infection) against symptomatic infection, bias compared to adjusted VE (controlling for prior infection) against symptomatic infection, the percentage of simulations with a bias less than or equal to 6 percentage points (pp), 8 pp, and VE estimate less than zero (negative VE) for 1-2 months since the last vaccination.

Supplementary Fig. 9 Notes: Estimates are generated from a meta-regression of aggregated results from 768 simulation conditions (each of which were summarized from 1,000 simulations) after controlling for all other simulation parameters. Bias is computed as the difference between the unadjusted VE estimate compared to the VE estimate adjusted for prior infection. Estimates are presented as the marginal mean (as dots) +/- the 95% confidence interval (represented by solid lines) that are a product of the standard error and normal distribution quantiles. The bars may be narrower than the dot and not visible. The dashed line and shaded region in the VE column represent the overall mean and the 95% confidence interval, respectively, from Fig. 1. In the Bias column, the dashed line and shaded region represent the overall mean and the 95% confidence interval, respectively, from Supplementary Fig. 3.

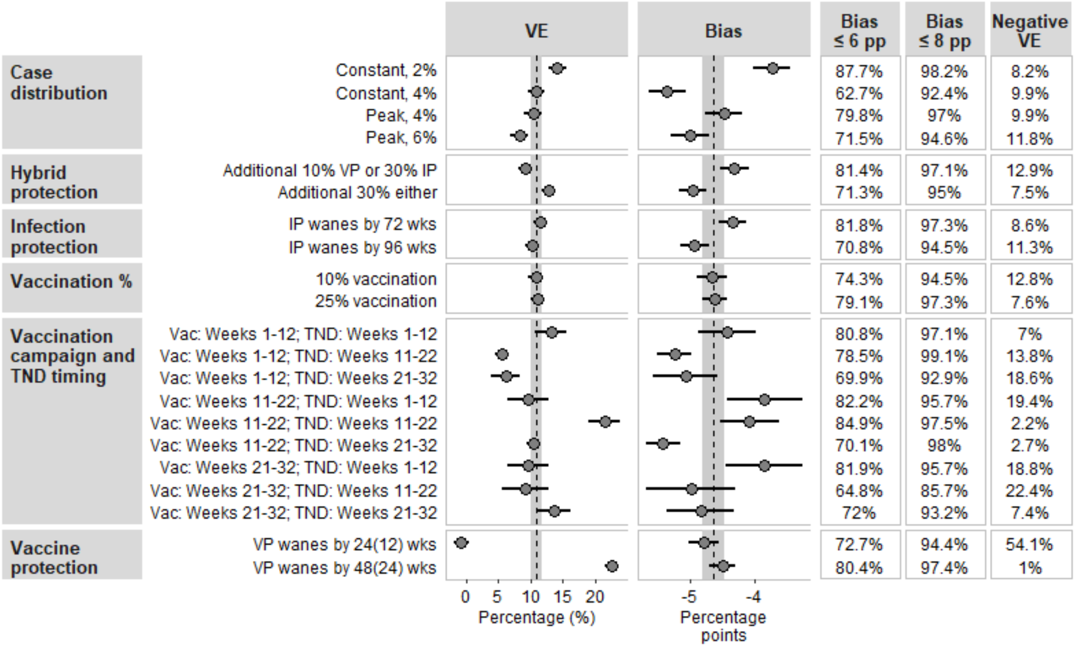

Supplementary Fig. 10: Plot of estimated marginal means of unadjusted VE (not controlling for prior infection) against symptomatic infection, bias compared to adjusted VE (controlling for prior infection) against symptomatic infection, the percentage of simulations with a bias less than or equal to 6 percentage points (pp), 8 pp, and VE estimate less than zero (negative VE) for 3-4 months since the last vaccination.

Supplementary Fig. 10 Notes: Estimates are generated from a meta-regression of aggregated results from 768 simulation conditions (each of which were summarized from 1,000 simulations) after controlling for all other simulation parameters. Bias is computed as the difference between the unadjusted VE estimate compared to the VE estimate adjusted for prior infection. Estimates are presented as the marginal mean (as dots) +/- the 95% confidence interval (represented by solid lines) that are a product of the standard error and normal distribution quantiles. The bars may be narrower than the dot and not visible. The dashed line and shaded region in the VE column represent the overall mean and the 95% confidence interval, respectively, from Fig. 1. In the Bias column, the dashed line and shaded region represent the overall mean and the 95% confidence interval, respectively, from Supplementary Fig. 3.

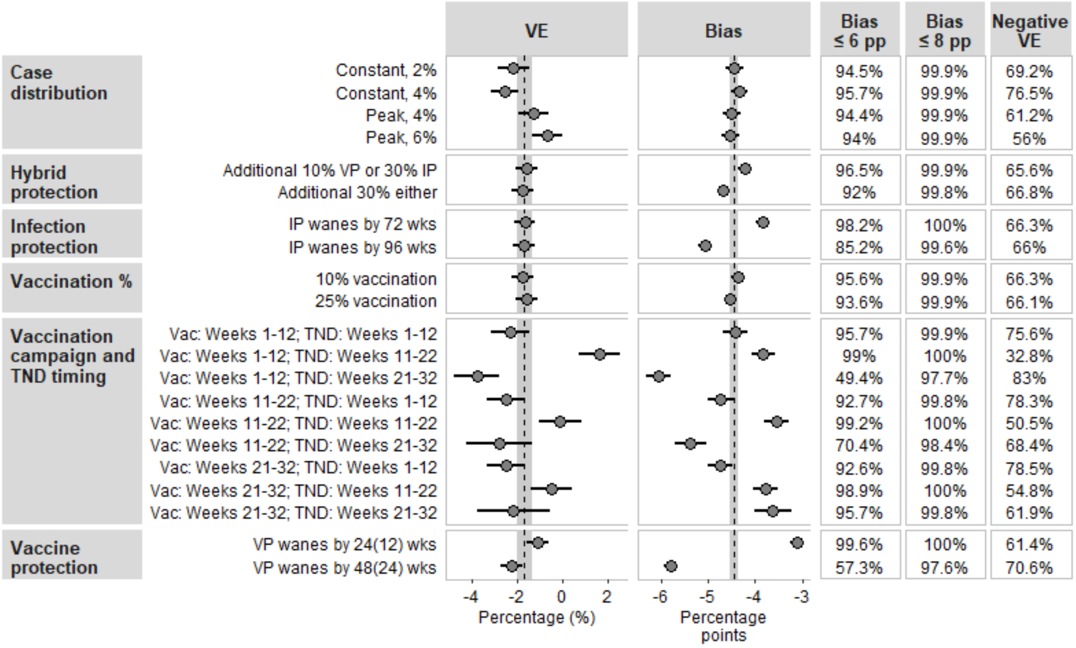

Supplementary Fig. 11: Plot of estimated marginal means of unadjusted VE (not controlling for prior infection) against symptomatic infection, bias compared to adjusted VE (controlling for prior infection) against symptomatic infection, the percentage of simulations with a bias less than or equal to 6 percentage points (pp), 8 pp, and VE estimate less than zero (negative VE) for 5-11 months since the last vaccination.

Supplementary Fig. 11 Notes: Estimates are generated from a meta-regression of aggregated results from 768 simulation conditions (each of which were summarized from 1,000 simulations) after controlling for all other simulation parameters. Bias is computed as the difference between the unadjusted VE estimate compared to the VE estimate adjusted for prior infection. Estimates are presented as the marginal mean (as dots) +/- the 95% confidence interval (represented by solid lines) that are a product of the standard error and normal distribution quantiles. The bars may be narrower than the dot and not visible. The dashed line and shaded region in the VE column represent the overall mean and the 95% confidence interval, respectively, from Fig. 1. In the Bias column, the dashed line and shaded region represent the overall mean and the 95% confidence interval, respectively, from Supplementary Fig. 3.

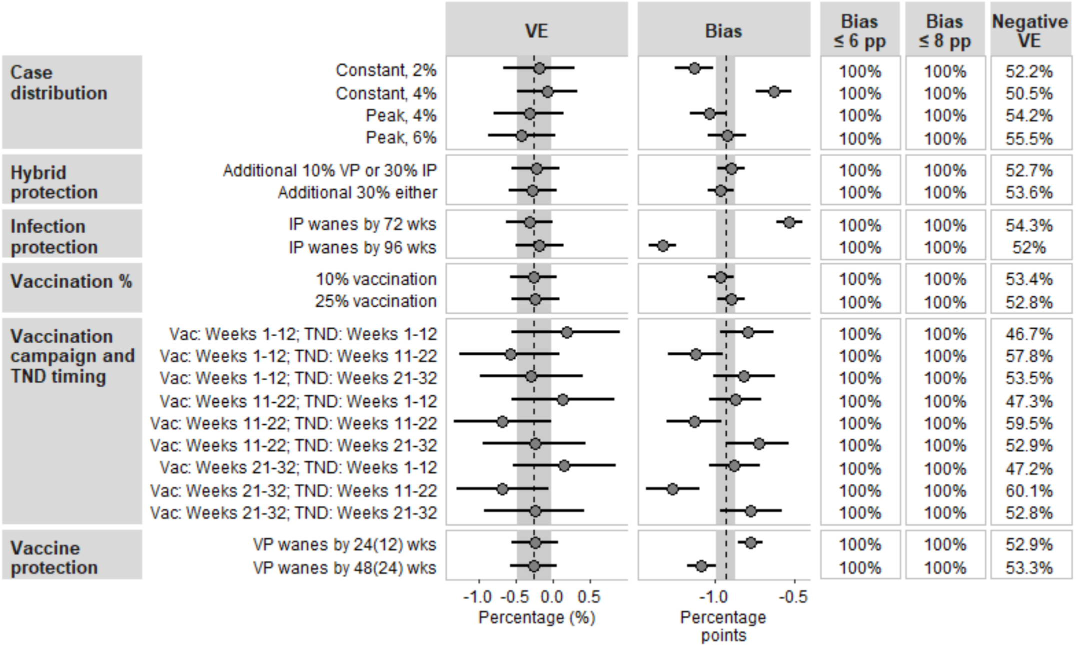

Supplementary Fig. 12: Plot of estimated marginal means of unadjusted VE (not controlling for prior infection) against symptomatic infection, bias compared to adjusted VE (controlling for prior infection) against symptomatic infection, the percentage of simulations with a bias less than or equal to 6 percentage points (pp), 8 pp, and VE estimate less than zero (negative VE) for 12 or more months since the last vaccination.

Supplementary Fig. 12 Notes: Estimates are generated from a meta-regression of aggregated results from 768 simulation conditions (each of which were summarized from 1,000 simulations) after controlling for all other simulation parameters. Bias is computed as the difference between the unadjusted VE estimate compared to the VE estimate adjusted for prior infection. Estimates are presented as the marginal mean (as dots) +/- the 95% confidence interval (represented by solid lines) that are a product of the standard error and normal distribution quantiles. The bars may be narrower than the dot and not visible. The dashed line and shaded region in the VE column represent the overall mean and the 95% confidence interval, respectively, from Fig. 1. In the Bias column, the dashed line and shaded region represent the overall mean and the 95% confidence interval, respectively, from Supplementary Fig. 3.

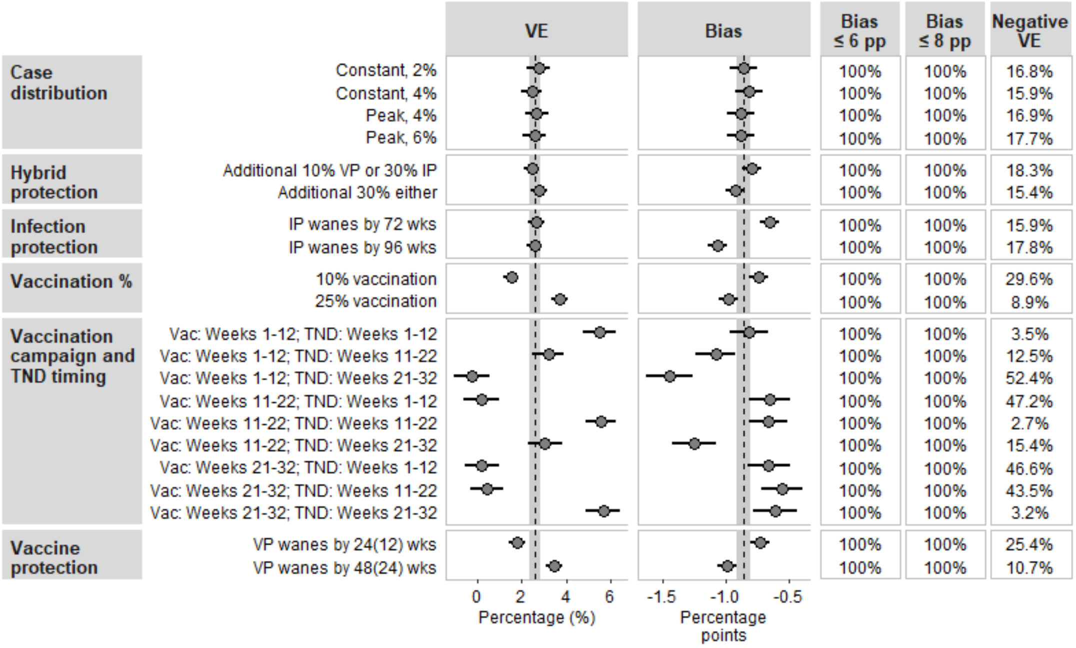

Supplementary Fig. 13: Plot of estimated marginal means of unadjusted VE (not controlling for prior infection) against symptomatic infection, bias compared to adjusted VE (controlling for prior infection) against symptomatic infection, the percentage of simulations with a bias less than or equal to 6 percentage points (pp), 8 pp, and VE estimate less than zero (negative VE) for 2 vaccination doses.

Supplementary Fig. 13 Notes: Estimates are generated from a meta-regression of aggregated results from 768 simulation conditions (each of which were summarized from 1,000 simulations) after controlling for all other simulation parameters. Bias is computed as the difference between the unadjusted VE estimate compared to the VE estimate adjusted for prior infection. Estimates are presented as the marginal mean (as dots) +/- the 95% confidence interval (represented by solid lines) that are a product of the standard error and normal distribution quantiles. The bars may be narrower than the dot and not visible. The dashed line and shaded region in the VE column represent the overall mean and the 95% confidence interval, respectively, from Fig. 1. In the Bias column, the dashed line and shaded region represent the overall mean and the 95% confidence interval, respectively, from Supplementary Fig. 3.

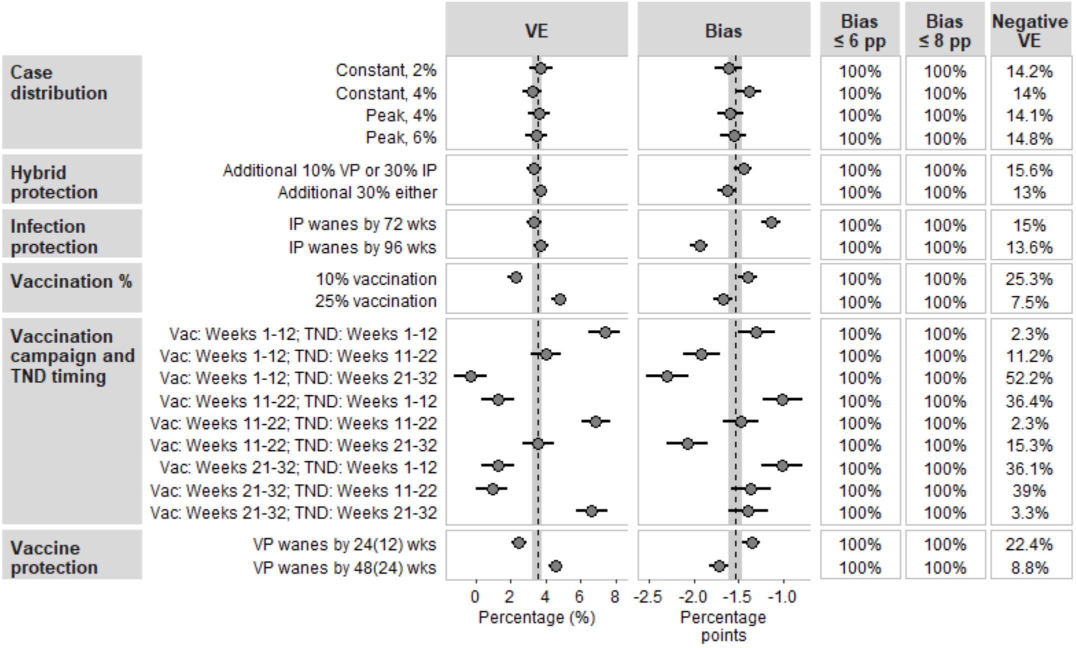

Supplementary Fig. 14: Plot of estimated marginal means of unadjusted VE (not controlling for prior infection) against symptomatic infection, bias compared to adjusted VE (controlling for prior infection) against symptomatic infection, the percentage of simulations with a bias less than or equal to 6 percentage points (pp), 8 pp, and VE estimate less than zero (negative VE) for 3 vaccination doses.

Supplementary Fig. 14 Notes: Estimates are generated from a meta-regression of aggregated results from 768 simulation conditions (each of which were summarized from 1,000 simulations) after controlling for all other simulation parameters. Bias is computed as the difference between the unadjusted VE estimate compared to the VE estimate adjusted for prior infection. Estimates are presented as the marginal mean (as dots) +/- the 95% confidence interval (represented by solid lines) that are a product of the standard error and normal distribution quantiles. The bars may be narrower than the dot and not visible. The dashed line and shaded region in the VE column represent the overall mean and the 95% confidence interval, respectively, from Fig. 1. In the Bias column, the dashed line and shaded region represent the overall mean and the 95% confidence interval, respectively, from Supplementary Fig. 3.

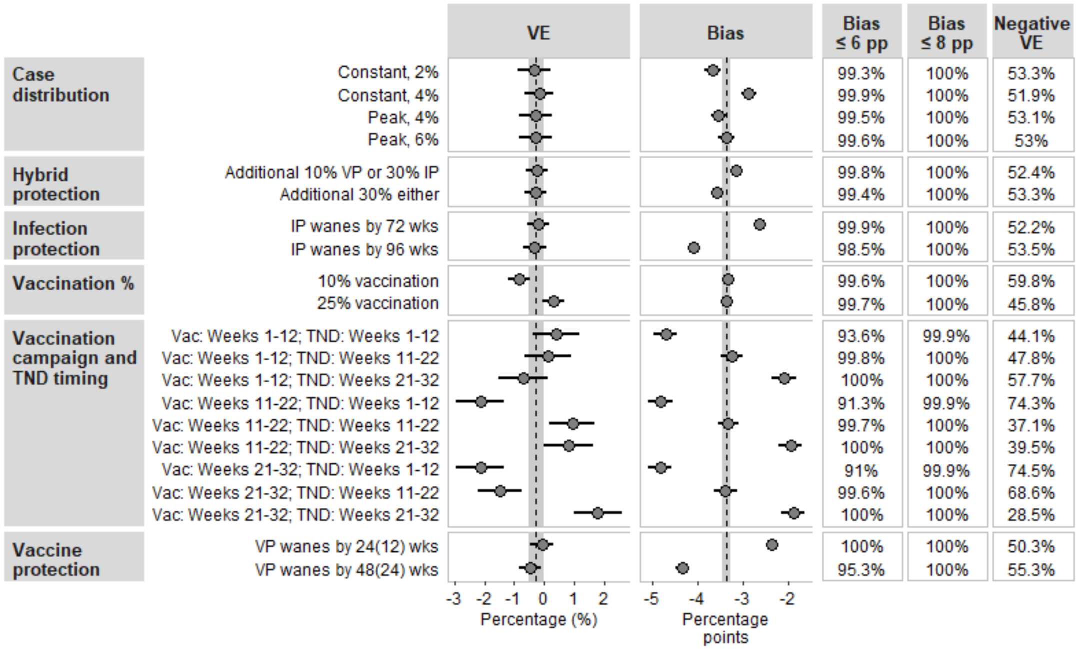

Supplementary Fig. 15: Plot of estimated marginal means of unadjusted VE (not controlling for prior infection) against symptomatic infection, bias compared to adjusted VE (controlling for prior infection) against symptomatic infection, the percentage of simulations with a bias less than or equal to 6 percentage points (pp), 8 pp, and VE estimate less than zero (negative VE) for 4 vaccination doses.

Supplementary Fig. 15 Notes: Estimates are generated from a meta-regression of aggregated results from 768 simulation conditions (each of which were summarized from 1,000 simulations) after controlling for all other simulation parameters. Bias is computed as the difference between the unadjusted VE estimate compared to the VE estimate adjusted for prior infection. Estimates are presented as the marginal mean (as dots) +/- the 95% confidence interval (represented by solid lines) that are a product of the standard error and normal distribution quantiles. The bars may be narrower than the dot and not visible. The dashed line and shaded region in the VE column represent the overall mean and the 95% confidence interval, respectively, from Fig. 1. In the Bias column, the dashed line and shaded region represent the overall mean and the 95% confidence interval, respectively, from Supplementary Fig. 3.

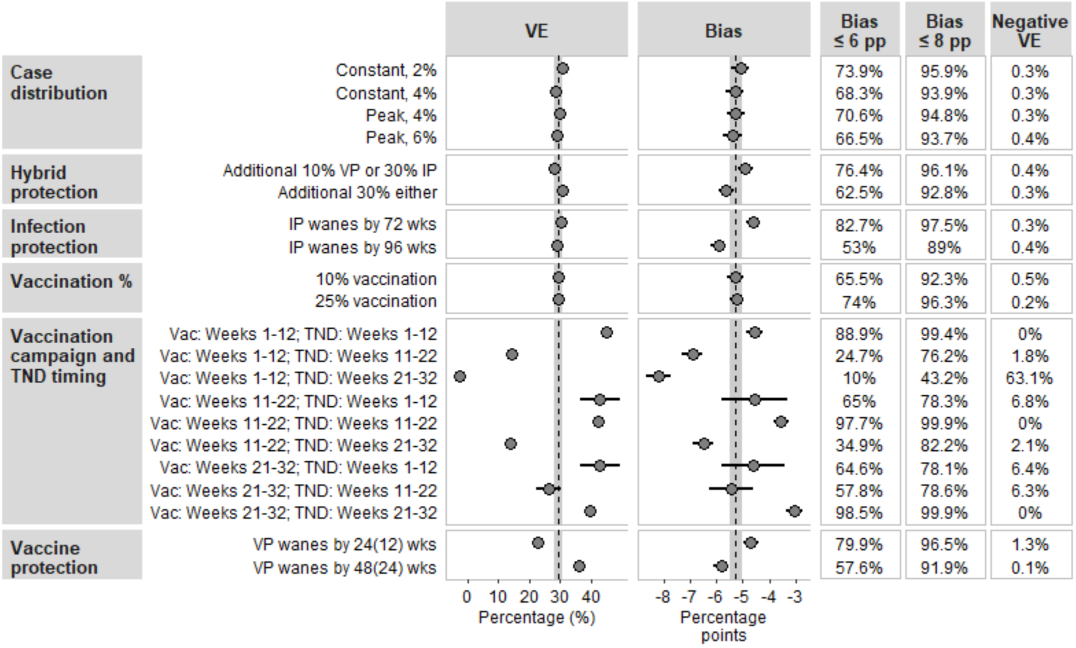

Supplementary Fig. 16: Plot of estimated marginal means of unadjusted VE (not controlling for prior infection) against symptomatic infection, bias compared to adjusted VE (controlling for prior infection) against symptomatic infection, the percentage of simulations with a bias less than or equal to 6 percentage points (pp), 8 pp, and VE estimate less than zero (negative VE) for 5 vaccination doses.

Supplementary Fig. 16 Notes: Estimates are generated from a meta-regression of aggregated results from 768 simulation conditions (each of which were summarized from 1,000 simulations) after controlling for all other simulation parameters. Bias is computed as the difference between the unadjusted VE estimate compared to the VE estimate adjusted for prior infection. Estimates are presented as the marginal mean (as dots) +/- the 95% confidence interval (represented by solid lines) that are a product of the standard error and normal distribution quantiles. The bars may be narrower than the dot and not visible. The dashed line and shaded region in the VE column represent the overall mean and the 95% confidence interval, respectively, from Fig. 1. In the Bias column, the dashed line and shaded region represent the overall mean and the 95% confidence interval, respectively, from Supplementary Fig. 3.

VE against severe disease

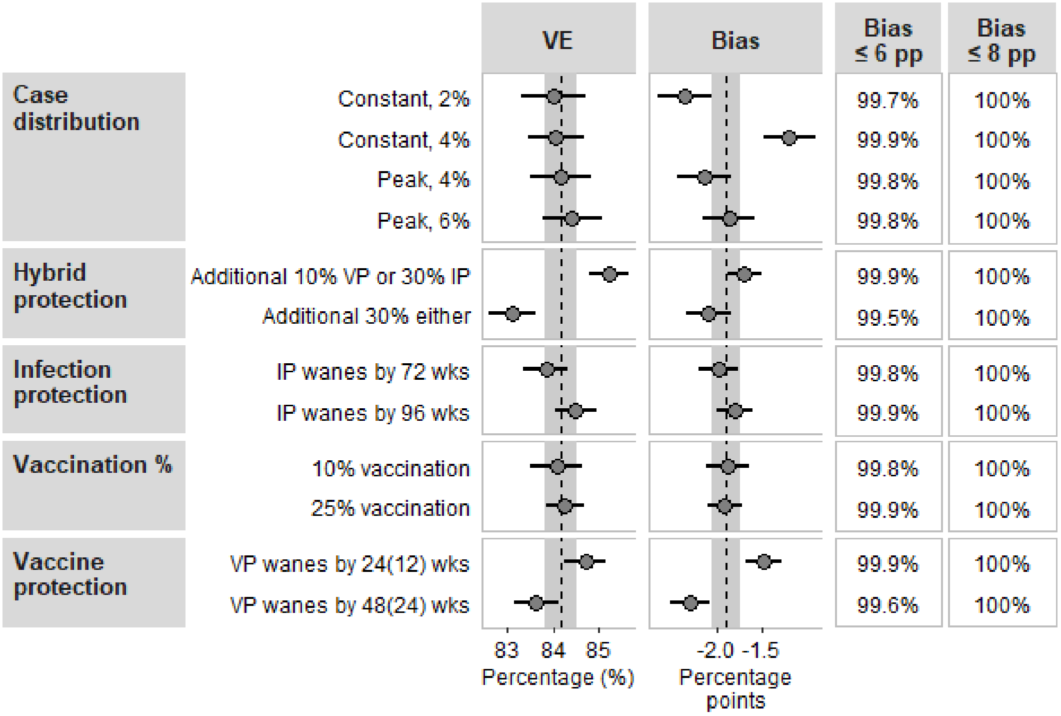

Supplementary Fig. 17: Plot of estimated marginal means of unadjusted VE (not controlling for prior infection) against severe disease, bias compared to adjusted VE (controlling for prior infection), the percentage of simulations with a bias less than or equal to 6 percentage points (pp), or 8 pp for vaccination at any time during the analytic period.

Supplementary Fig. 17 Notes: Estimates are generated from a meta-regression of aggregated results from 768 simulation conditions (each of which were summarized from 1,000 simulations) after controlling for all other simulation parameters. Bias is computed as the difference between the unadjusted VE estimate compared to the VE estimate adjusted for prior infection. Estimates are presented as the marginal mean (as dots) +/- the 95% confidence interval (represented by solid lines) that are a product of the standard error and normal distribution quantiles. The dashed line and shaded region in the VE column represent the overall mean and the 95% confidence interval, respectively, from Fig. 1. In the Bias column, the dashed line and shaded region represent the overall mean and the 95% confidence interval, respectively, from Supplementary Fig. 3.

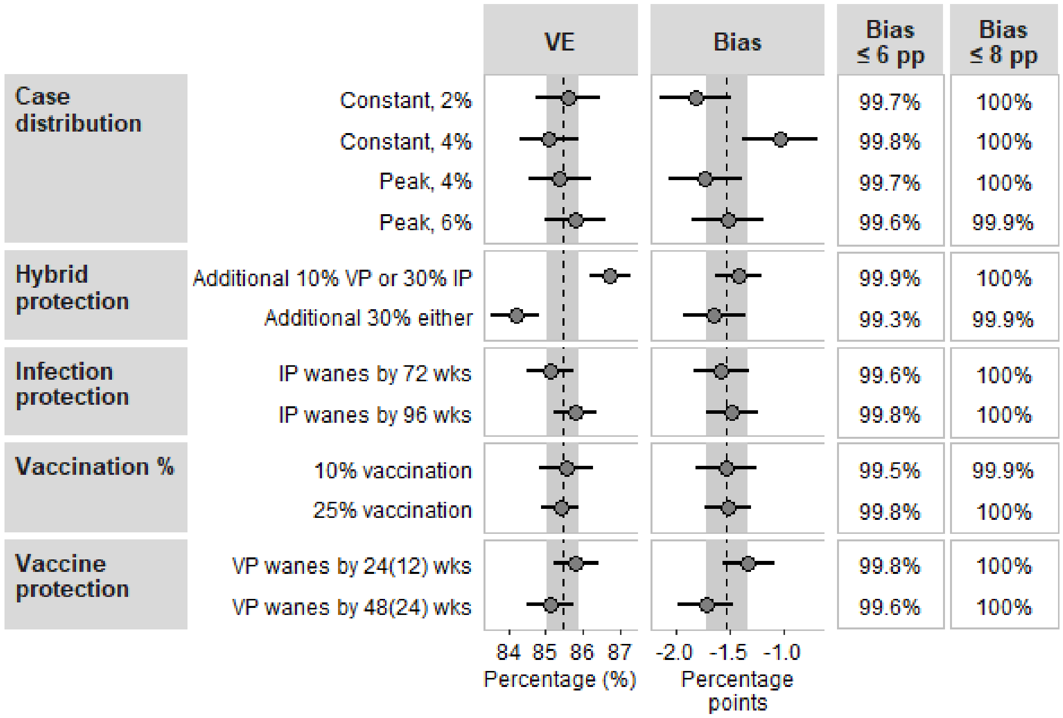

Supplementary Fig. 18: Plot of estimated marginal means of unadjusted VE (not controlling for prior infection) against severe disease, bias compared to adjusted VE (controlling for prior infection), the percentage of simulations with a bias less than or equal to 6 percentage points (pp), or 8 pp for vaccination in the previous 2 months.

Supplementary Fig. 18 Notes: Estimates are generated from a meta-regression of aggregated results from 768 simulation conditions (each of which were summarized from 1,000 simulations) after controlling for all other simulation parameters. Bias is computed as the difference between the unadjusted VE estimate compared to the VE estimate adjusted for prior infection. Estimates are presented as the marginal mean (as dots) +/- the 95% confidence interval (represented by solid lines) that are a product of the standard error and normal distribution quantiles. The dashed line and shaded region in the VE column represent the overall mean and the 95% confidence interval, respectively, from Fig. 1. In the Bias column, the dashed line and shaded region represent the overall mean and the 95% confidence interval, respectively, from Supplementary Fig. 3.

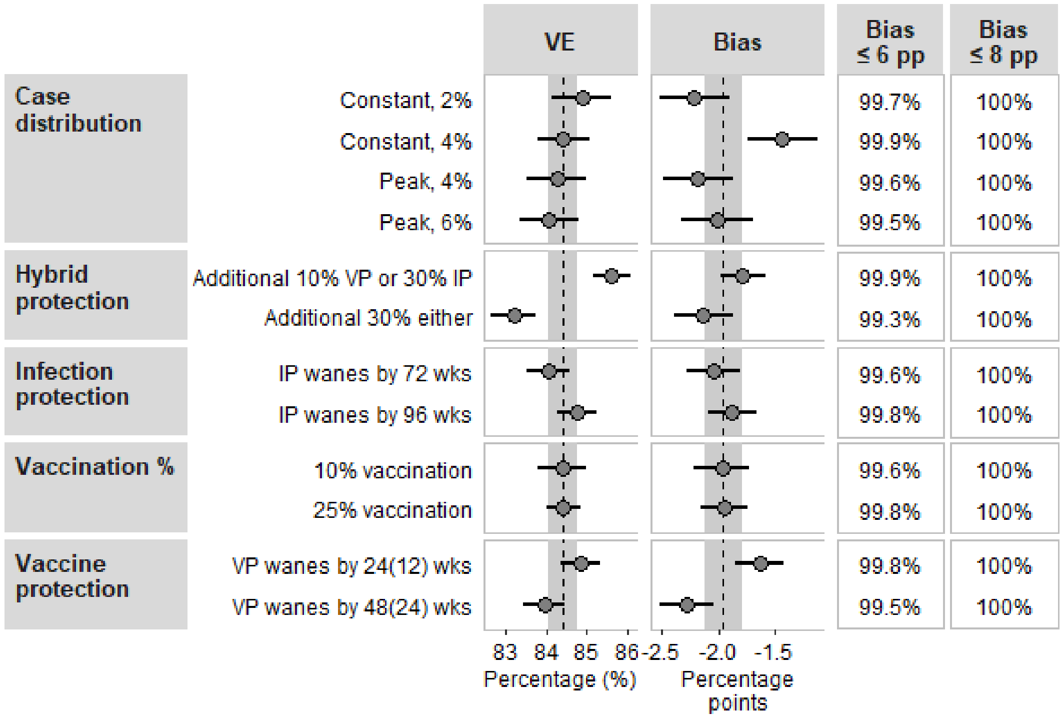

Supplementary Fig. 19: Plot of estimated marginal means of unadjusted VE (not controlling for prior infection) against severe disease, bias compared to adjusted VE (controlling for prior infection), the percentage of simulations with a bias less than or equal to 6 percentage points (pp), or 8 pp for vaccination in the previous 4 months.

Supplementary Fig. 19 Notes: Estimates are generated from a meta-regression of aggregated results from 768 simulation conditions (each of which were summarized from 1,000 simulations) after controlling for all other simulation parameters. Bias is computed as the difference between the unadjusted VE estimate compared to the VE estimate adjusted for prior infection. Estimates are presented as the marginal mean (as dots) +/- the 95% confidence interval (represented by solid lines) that are a product of the standard error and normal distribution quantiles. The dashed line and shaded region in the VE column represent the overall mean and the 95% confidence interval, respectively, from Fig. 1. In the Bias column, the dashed line and shaded region represent the overall mean and the 95% confidence interval, respectively, from Supplementary Fig. 3.

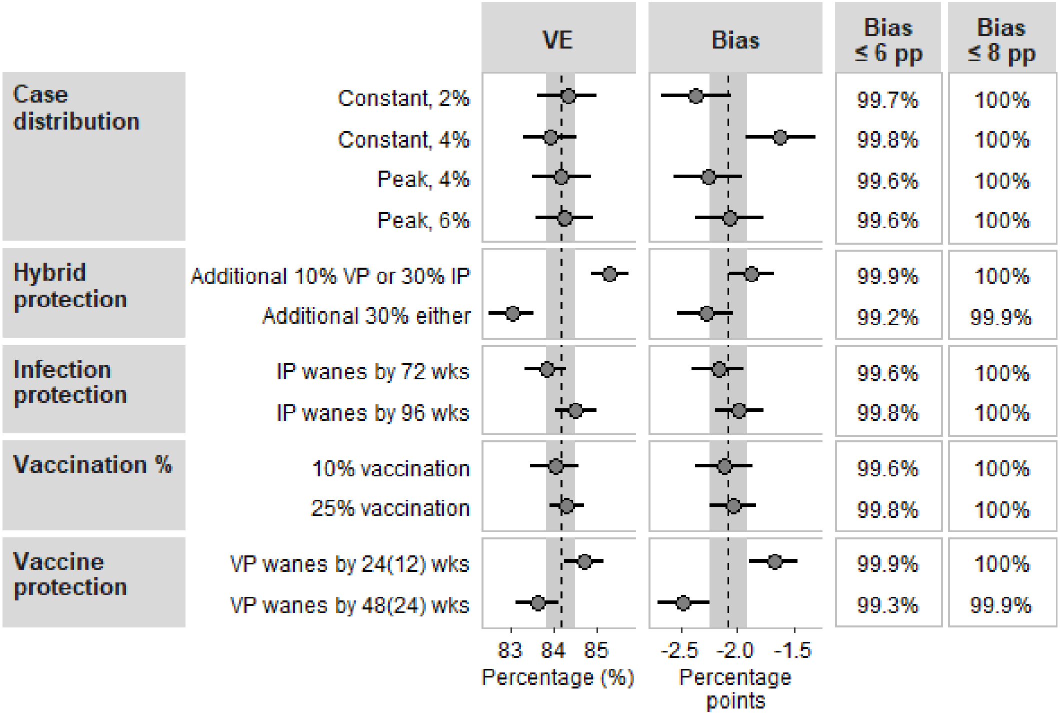

Supplementary Fig. 20: Plot of estimated marginal means of unadjusted VE (not controlling for prior infection) against severe disease, bias compared to adjusted VE (controlling for prior infection), the percentage of simulations with a bias less than or equal to 6 percentage points (pp), or 8 pp for vaccination in the previous 5 months.

Supplementary Fig. 20 Notes: Estimates are generated from a meta-regression of aggregated results from 768 simulation conditions (each of which were summarized from 1,000 simulations) after controlling for all other simulation parameters. Bias is computed as the difference between the unadjusted VE estimate compared to the VE estimate adjusted for prior infection. Estimates are presented as the marginal mean (as dots) +/- the 95% confidence interval (represented by solid lines) that are a product of the standard error and normal distribution quantiles. The dashed line and shaded region in the VE column represent the overall mean and the 95% confidence interval, respectively, from Fig. 1. In the Bias column, the dashed line and shaded region represent the overall mean and the 95% confidence interval, respectively, from Supplementary Fig. 3.

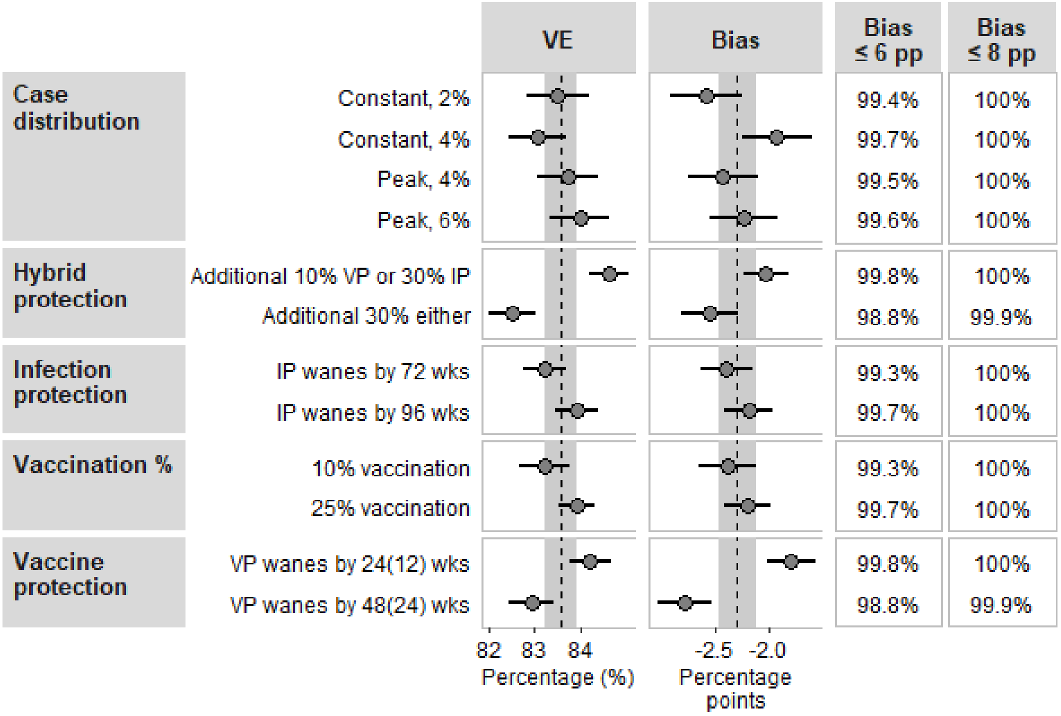

Supplementary Fig. 21: Plot of estimated marginal means of unadjusted VE (not controlling for prior infection) against severe disease, bias compared to adjusted VE (controlling for prior infection), the percentage of simulations with a bias less than or equal to 6 percentage points (pp), or 8 pp for vaccination in the previous 6 months.

Supplementary Fig. 21 Notes: Estimates are generated from a meta-regression of aggregated results from 768 simulation conditions (each of which were summarized from 1,000 simulations) after controlling for all other simulation parameters. Bias is computed as the difference between the unadjusted VE estimate compared to the VE estimate adjusted for prior infection. Estimates are presented as the marginal mean (as dots) +/- the 95% confidence interval (represented by solid lines) that are a product of the standard error and normal distribution quantiles. The dashed line and shaded region in the VE column represent the overall mean and the 95% confidence interval, respectively, from Fig. 1. In the Bias column, the dashed line and shaded region represent the overall mean and the 95% confidence interval, respectively, from Supplementary Fig. 3.

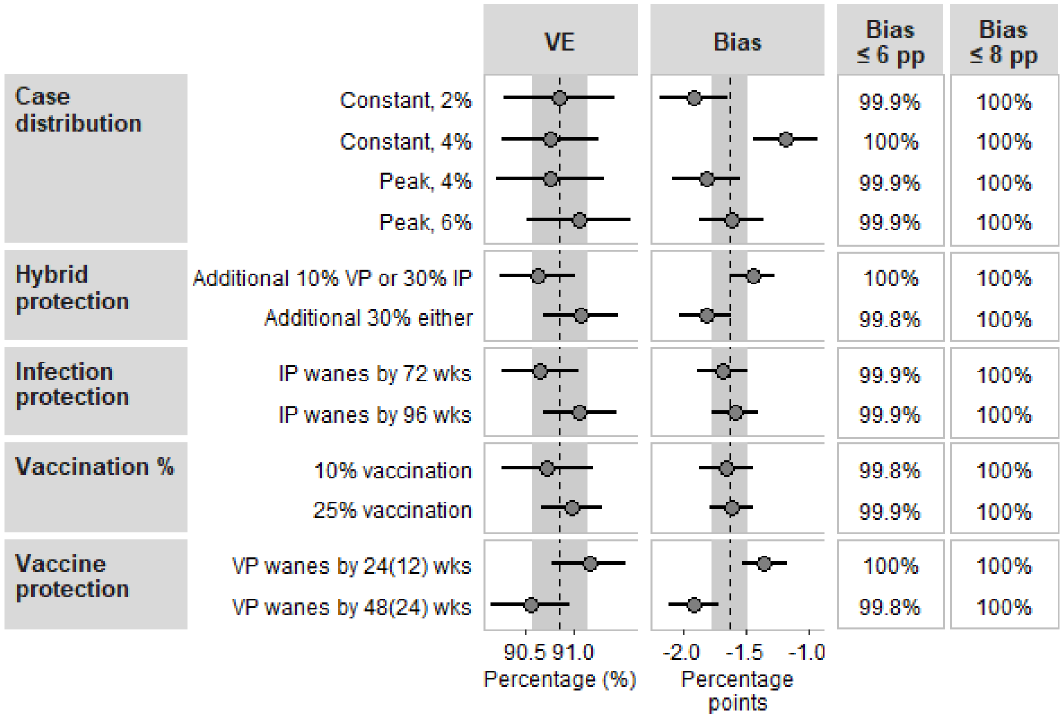

Supplementary Fig. 22: Plot of estimated marginal means of unadjusted VE (not controlling for prior infection) against severe disease, bias compared to adjusted VE (controlling for prior infection), the percentage of simulations with a bias less than or equal to 6 percentage points (pp), or 8 pp for 1-2 months since the last vaccination.

Supplementary Fig. 22 Notes: Estimates are generated from a meta-regression of aggregated results from 768 simulation conditions (each of which were summarized from 1,000 simulations) after controlling for all other simulation parameters. Bias is computed as the difference between the unadjusted VE estimate compared to the VE estimate adjusted for prior infection. Estimates are presented as the marginal mean (as dots) +/- the 95% confidence interval (represented by solid lines) that are a product of the standard error and normal distribution quantiles. The dashed line and shaded region in the VE column represent the overall mean and the 95% confidence interval, respectively, from Fig. 1. In the Bias column, the dashed line and shaded region represent the overall mean and the 95% confidence interval, respectively, from Supplementary Fig. 3.

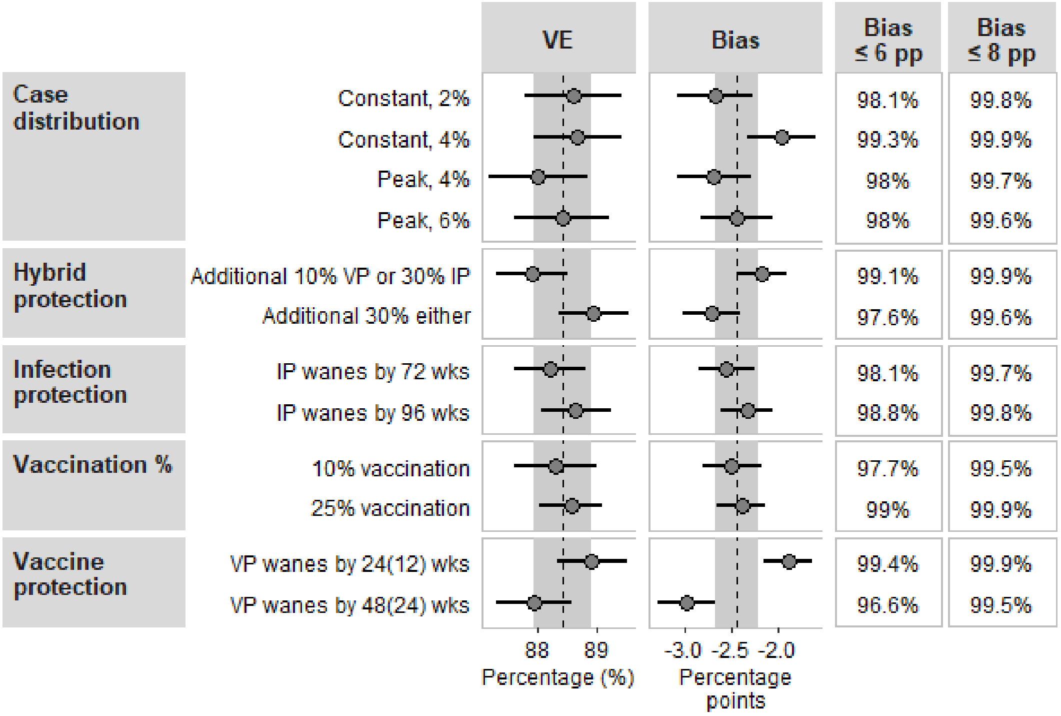

Supplementary Fig. 23: Plot of estimated marginal means of unadjusted VE (not controlling for prior infection) against severe disease, bias compared to adjusted VE (controlling for prior infection), the percentage of simulations with a bias less than or equal to 6 percentage points (pp), or 8 pp for 3-4 months since the last vaccination.

Supplementary Fig. 23 Notes: Estimates are generated from a meta-regression of aggregated results from 768 simulation conditions (each of which were summarized from 1,000 simulations) after controlling for all other simulation parameters. Bias is computed as the difference between the unadjusted VE estimate compared to the VE estimate adjusted for prior infection. Estimates are presented as the marginal mean (as dots) +/- the 95% confidence interval (represented by solid lines) that are a product of the standard error and normal distribution quantiles. The dashed line and shaded region in the VE column represent the overall mean and the 95% confidence interval, respectively, from Fig. 1. In the Bias column, the dashed line and shaded region represent the overall mean and the 95% confidence interval, respectively, from Supplementary Fig. 3.

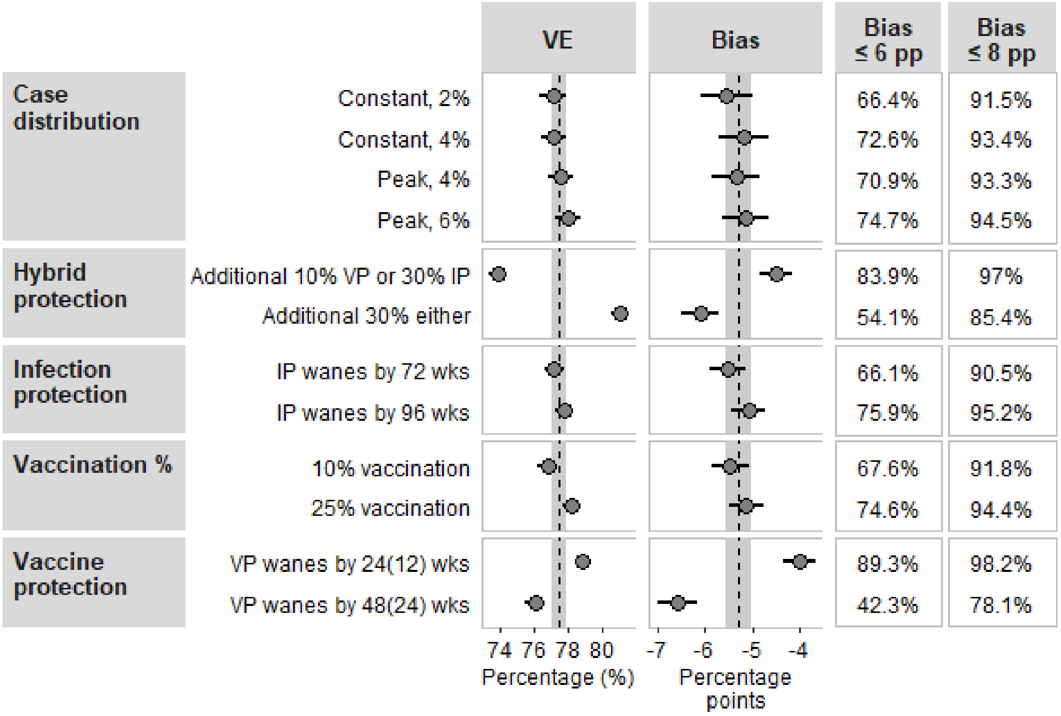

Supplementary Fig. 24: Plot of estimated marginal means of unadjusted VE (not controlling for prior infection) against severe disease, bias compared to adjusted VE (controlling for prior infection), the percentage of simulations with a bias less than or equal to 6 percentage points (pp), or 8 pp for 5-11 months since the last vaccination.

Supplementary Fig. 24 Notes: Estimates are generated from a meta-regression of aggregated results from 768 simulation conditions (each of which were summarized from 1,000 simulations) after controlling for all other simulation parameters. Bias is computed as the difference between the unadjusted VE estimate compared to the VE estimate adjusted for prior infection. Estimates are presented as the marginal mean (as dots) +/- the 95% confidence interval (represented by solid lines) that are a product of the standard error and normal distribution quantiles. The dashed line and shaded region in the VE column represent the overall mean and the 95% confidence interval, respectively, from Fig. 1. In the Bias column, the dashed line and shaded region represent the overall mean and the 95% confidence interval, respectively, from Supplementary Fig. 3.

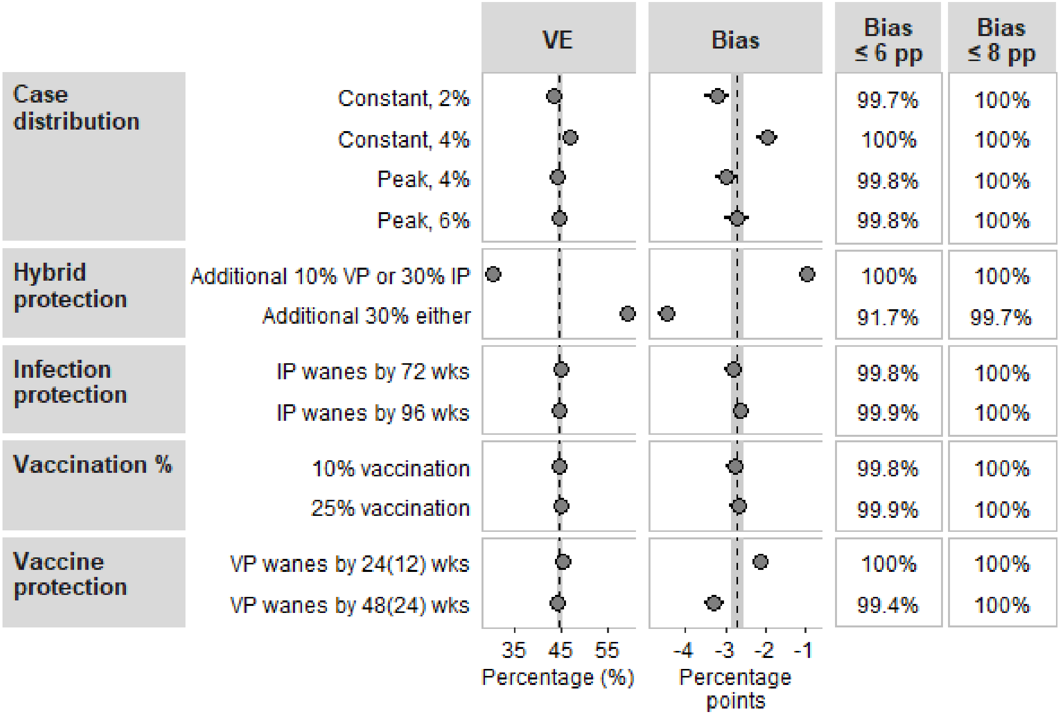

Supplementary Fig. 25: Plot of estimated marginal means of unadjusted VE (not controlling for prior infection) against severe disease, bias compared to adjusted VE (controlling for prior infection), the percentage of simulations with a bias less than or equal to 6 percentage points (pp), or 8 pp for 12 or more months since the last vaccination.

Supplementary Fig. 25 Notes: Estimates are generated from a meta-regression of aggregated results from 768 simulation conditions (each of which were summarized from 1,000 simulations) after controlling for all other simulation parameters. Bias is computed as the difference between the unadjusted VE estimate compared to the VE estimate adjusted for prior infection. Estimates are presented as the marginal mean (as dots) +/- the 95% confidence interval (represented by solid lines) that are a product of the standard error and normal distribution quantiles. The dashed line and shaded region in the VE column represent the overall mean and the 95% confidence interval, respectively, from Fig. 1. In the Bias column, the dashed line and shaded region represent the overall mean and the 95% confidence interval, respectively, from Supplementary Fig. 3.

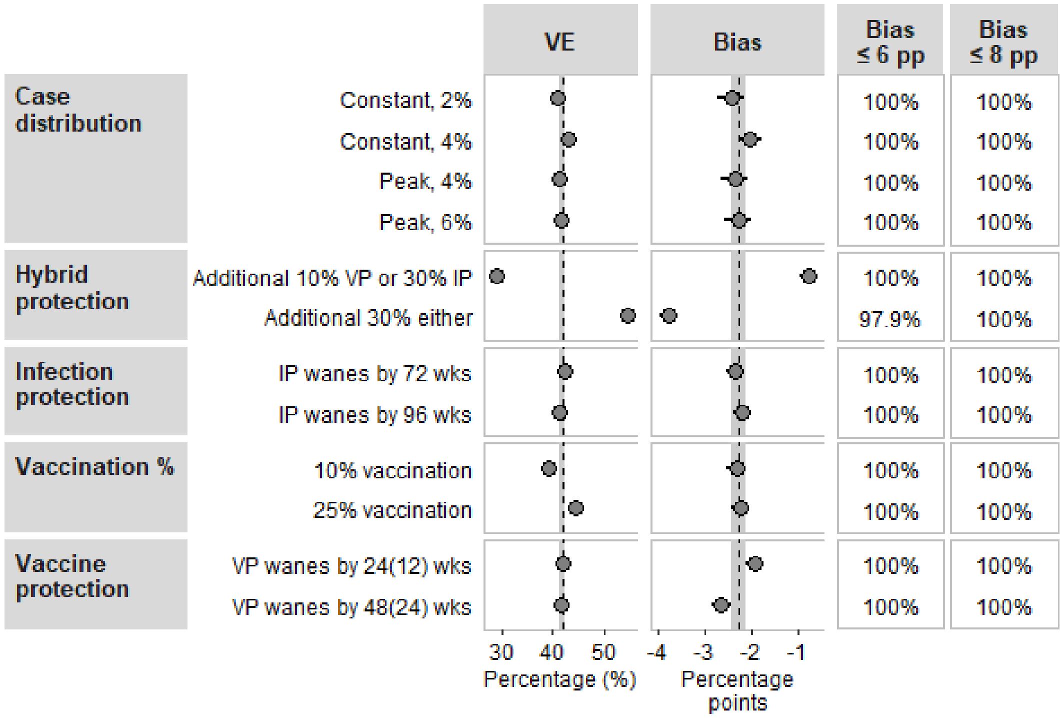

Supplementary Fig. 26: Plot of estimated marginal means of unadjusted VE (not controlling for prior infection) against severe disease, bias compared to adjusted VE (controlling for prior infection), the percentage of simulations with a bias less than or equal to 6 percentage points (pp), or 8 pp for 2 vaccination doses.

Supplementary Fig. 26 Notes: Estimates are generated from a meta-regression of aggregated results from 768 simulation conditions (each of which were summarized from 1,000 simulations) after controlling for all other simulation parameters. Bias is computed as the difference between the unadjusted VE estimate compared to the VE estimate adjusted for prior infection. Estimates are presented as the marginal mean (as dots) +/- the 95% confidence interval (represented by solid lines) that are a product of the standard error and normal distribution quantiles. The dashed line and shaded region in the VE column represent the overall mean and the 95% confidence interval, respectively, from Fig. 1. In the Bias column, the dashed line and shaded region represent the overall mean and the 95% confidence interval, respectively, from Supplementary Fig. 3.

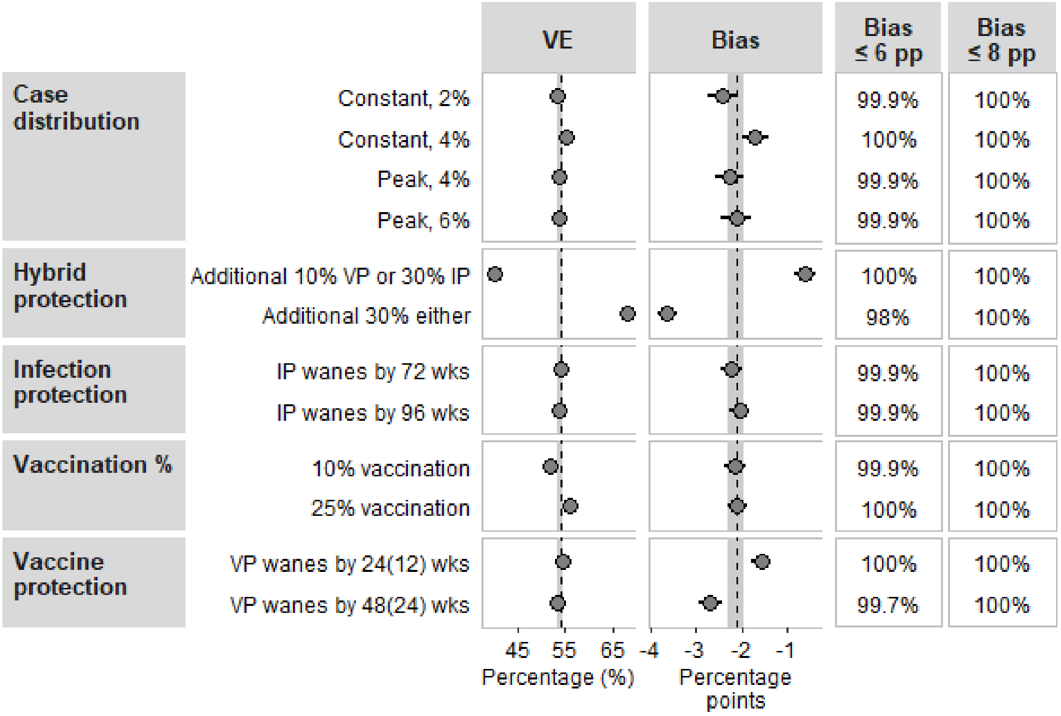

Supplementary Fig. 27: Plot of estimated marginal means of unadjusted VE (not controlling for prior infection) against severe disease, bias compared to adjusted VE (controlling for prior infection), the percentage of simulations with a bias less than or equal to 6 percentage points (pp), or 8 pp for 3 vaccination doses.

Supplementary Fig. 27 Notes: Estimates are generated from a meta-regression of aggregated results from 768 simulation conditions (each of which were summarized from 1,000 simulations) after controlling for all other simulation parameters. Bias is computed as the difference between the unadjusted VE estimate compared to the VE estimate adjusted for prior infection. Estimates are presented as the marginal mean (as dots) +/- the 95% confidence interval (represented by solid lines) that are a product of the standard error and normal distribution quantiles. The dashed line and shaded region in the VE column represent the overall mean and the 95% confidence interval, respectively, from Fig. 1. In the Bias column, the dashed line and shaded region represent the overall mean and the 95% confidence interval, respectively, from Supplementary Fig. 3.

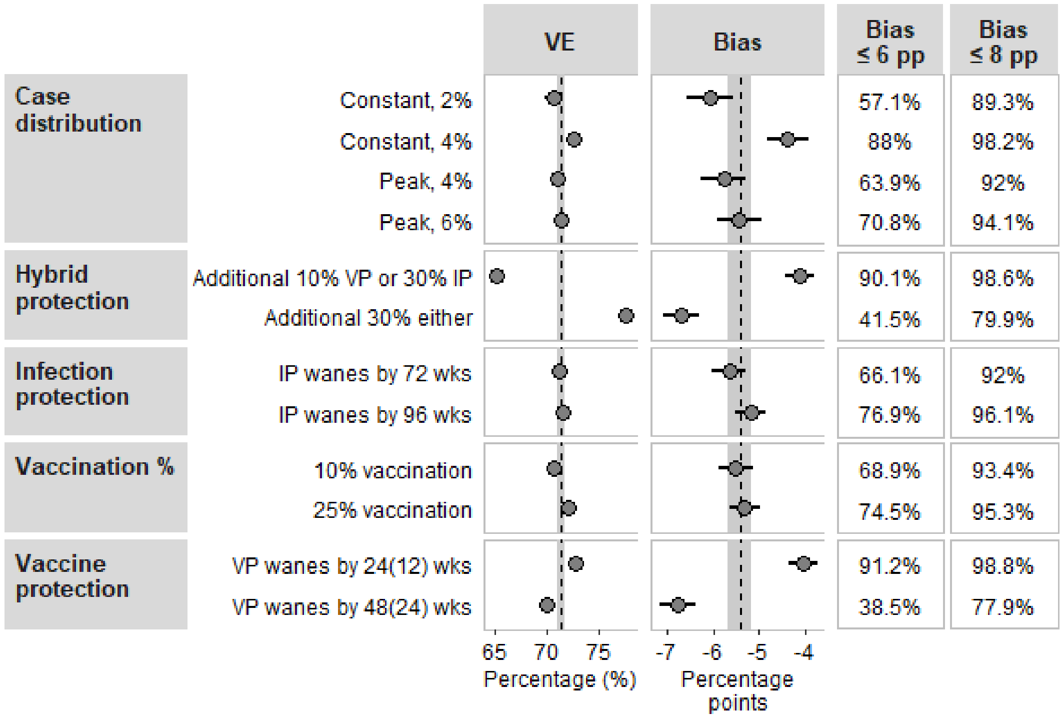

Supplementary Fig. 28: Plot of estimated marginal means of unadjusted VE (not controlling for prior infection) against severe disease, bias compared to adjusted VE (controlling for prior infection), the percentage of simulations with a bias less than or equal to 6 percentage points (pp), or 8 pp for 4 vaccination doses.

Supplementary Fig. 28 Notes: Estimates are generated from a meta-regression of aggregated results from 768 simulation conditions (each of which were summarized from 1,000 simulations) after controlling for all other simulation parameters. Bias is computed as the difference between the unadjusted VE estimate compared to the VE estimate adjusted for prior infection. Estimates are presented as the marginal mean (as dots) +/- the 95% confidence interval (represented by solid lines) that are a product of the standard error and normal distribution quantiles. The dashed line and shaded region in the VE column represent the overall mean and the 95% confidence interval, respectively, from Fig. 1. In the Bias column, the dashed line and shaded region represent the overall mean and the 95% confidence interval, respectively, from Supplementary Fig. 3.

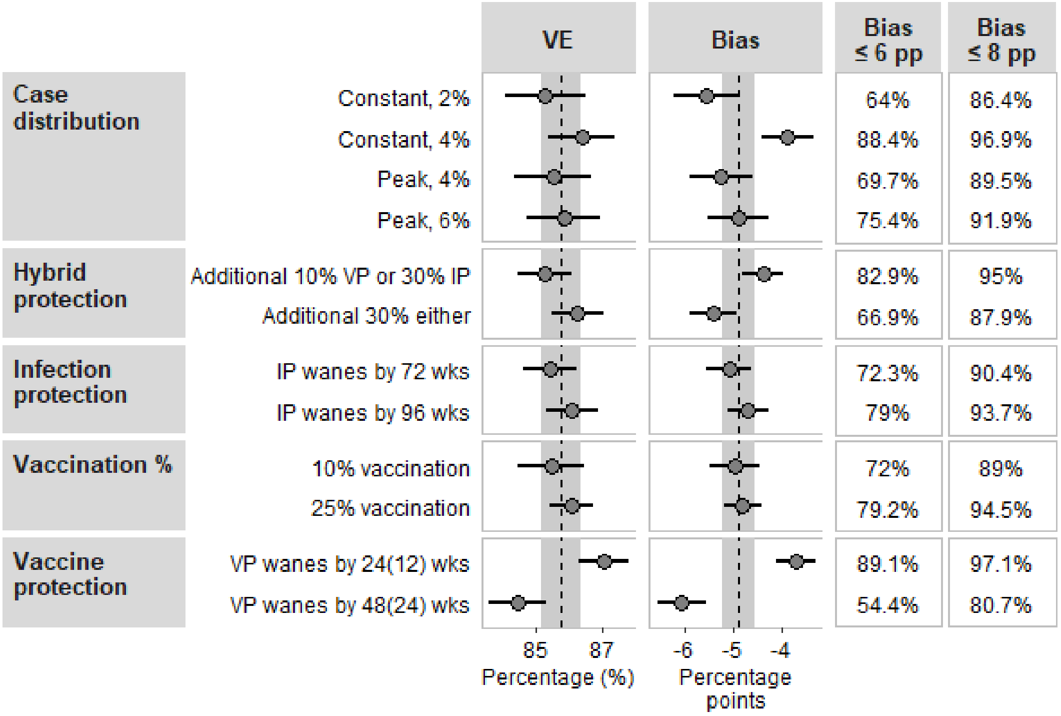

Supplementary Fig. 29: Plot of estimated marginal means of unadjusted VE (not controlling for prior infection) against severe disease, bias compared to adjusted VE (controlling for prior infection), the percentage of simulations with a bias less than or equal to 6 percentage points (pp), or 8 pp for 5 vaccination doses.

Supplementary Fig. 29 Notes: Estimates are generated from a meta-regression of aggregated results from 768 simulation conditions (each of which were summarized from 1,000 simulations) after controlling for all other simulation parameters. Bias is computed as the difference between the unadjusted VE estimate compared to the VE estimate adjusted for prior infection. Estimates are presented as the marginal mean (as dots) +/- the 95% confidence interval (represented by solid lines) that are a product of the standard error and normal distribution quantiles. The dashed line and shaded region in the VE column represent the overall mean and the 95% confidence interval, respectively, from Fig. 1. In the Bias column, the dashed line and shaded region represent the overall mean and the 95% confidence interval, respectively, from Supplementary Fig. 3.

Simulation methods

The simulation process was split into two parts. The goals of part one were to

1. create a vaccination and infection history for each person aged 18-49 years up to the week of 2023-05-07 to be used as covariates in modeling and
2. generate each person's protection level since a majority of people have existing protection against SARS-CoV-2 infection.

Part two then utilized the historical and protection information and applied those to a test-negative design (TND) to estimate vaccine effectiveness (VE) against symptomatic infection or severe disease.

Part 1: historical period

Probability of cases

Case count data from 60 U.S. jurisdictions [1] were summed by week to create weekly, national case counts. Weekly case counts were then divided by the 2020 U.S. population estimates to create provisional weekly probabilities of infection (Supplementary Fig. 30).

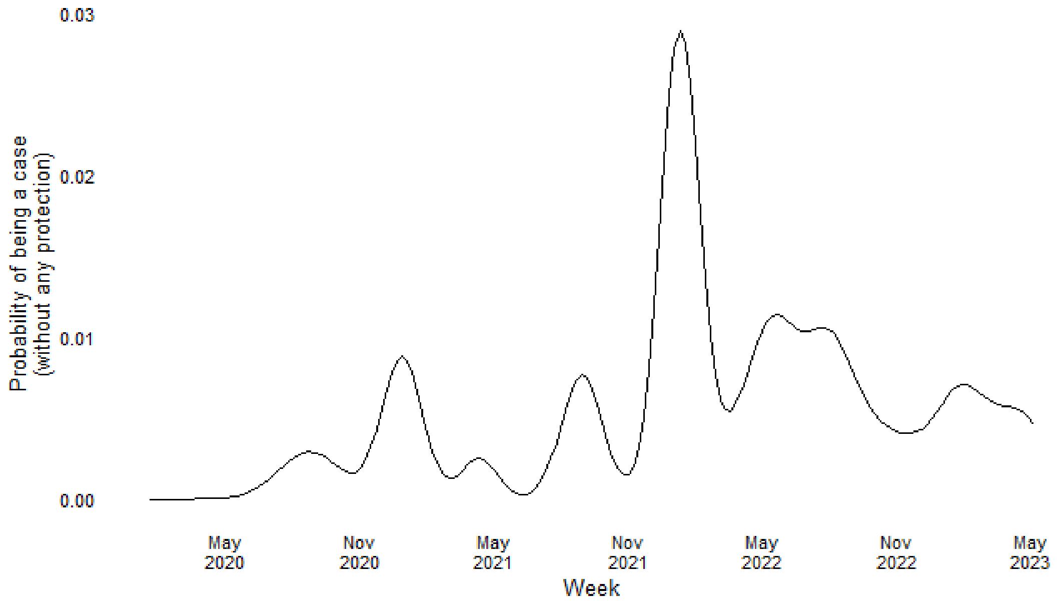

Supplementary Fig. 30: Weekly probability of infection based on U.S. jurisdictional case counts and 2020 U.S. population estimates.

Since case count data suffered from underreporting of infections that varied by time [2], we adjusted the distribution to be in line with seroprevalence studies that explored the underreporting [2] and found approximately 95%-98% of the population possessed at least one infection by the week of 2023-05-07 [3]. For each realized population, the

adjustment factor was varied allowing for some randomness in that factor. The distribution for the adjustment factor was $\mathcal{N}(\mu = 4, \sigma = 0.5)$ (Supplementary Fig. 31).

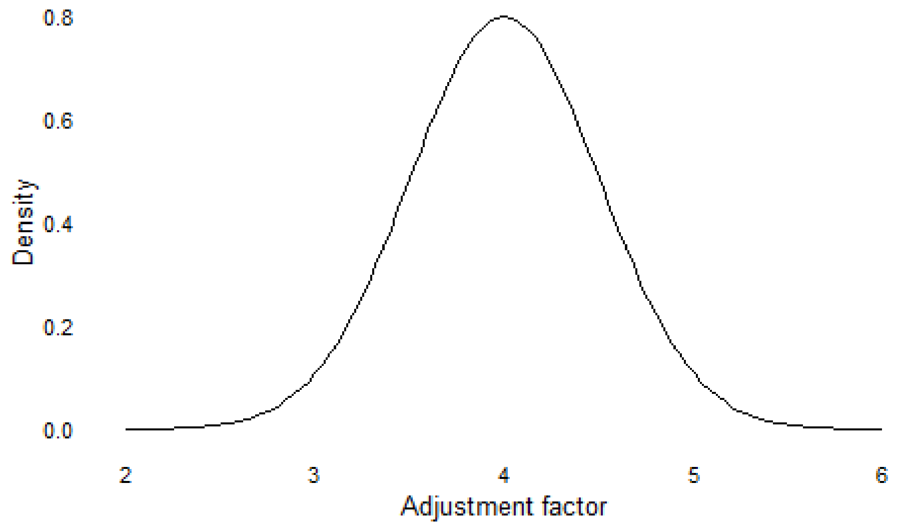

Supplementary Fig. 31: Distribution of the case distribution adjustment factor.

After accounting for the adjustment factor, the distribution became the weekly probability of infection without any protection. We used $\Pr(c_k)$ to denote the probability of infection in week k and plotted this below with the mean adjustment factor of 4 (Supplementary Fig. 32)

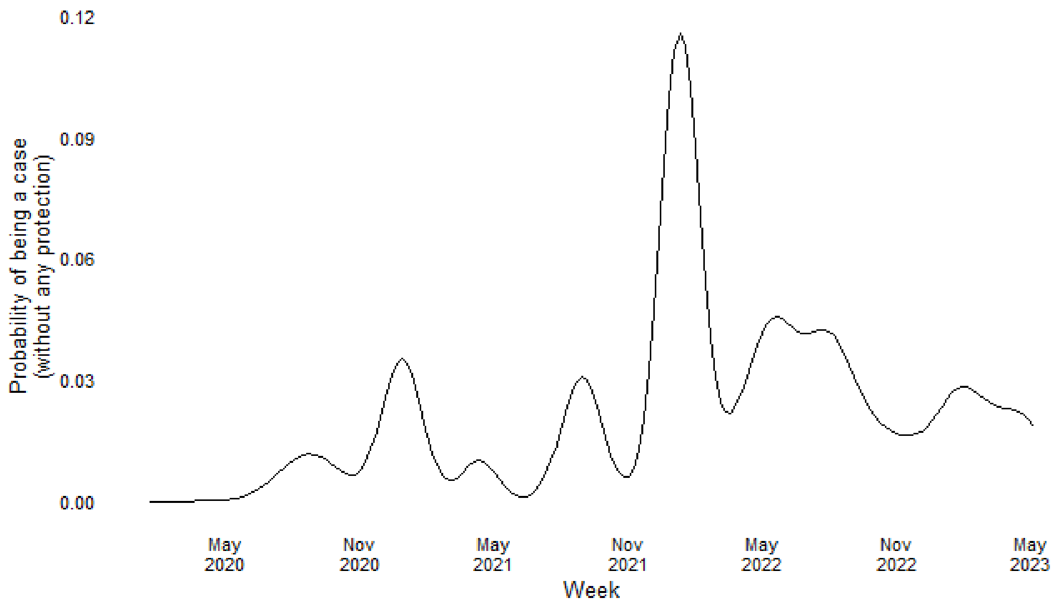

Supplementary Fig. 32: Weekly probability of infection with an adjustment factor of 4, the expected value of the adjustment factor distribution.

Probability of vaccination

The probability that person j got an additional vaccination dose in week k was conditional on their current dose. If $D_{j,k}$ was person j's number of vaccination doses at the beginning of week k and $D'_{j,k}$ the next dose for person j in week k, then $\Pr(D'_{j,k} \mid D_{j,k})$ was the likelihood of person j obtaining an additional vaccination dose in week k. In our simulations, we considered three distributions: $\Pr(D'_{j,k} = 2 \mid D_{j,k} = 0)$; $\Pr(D'_{j,k} = 3 \mid D_{j,k} = 2)$; and $\Pr(D'_{j,k} = 4 \mid D_{j,k} = 3)$

We used publicly available data of vaccination distributions for people aged 18-49 years by day [4] and converted them to weekly probabilities (Supplementary Fig. 33) for the vaccination distributions.

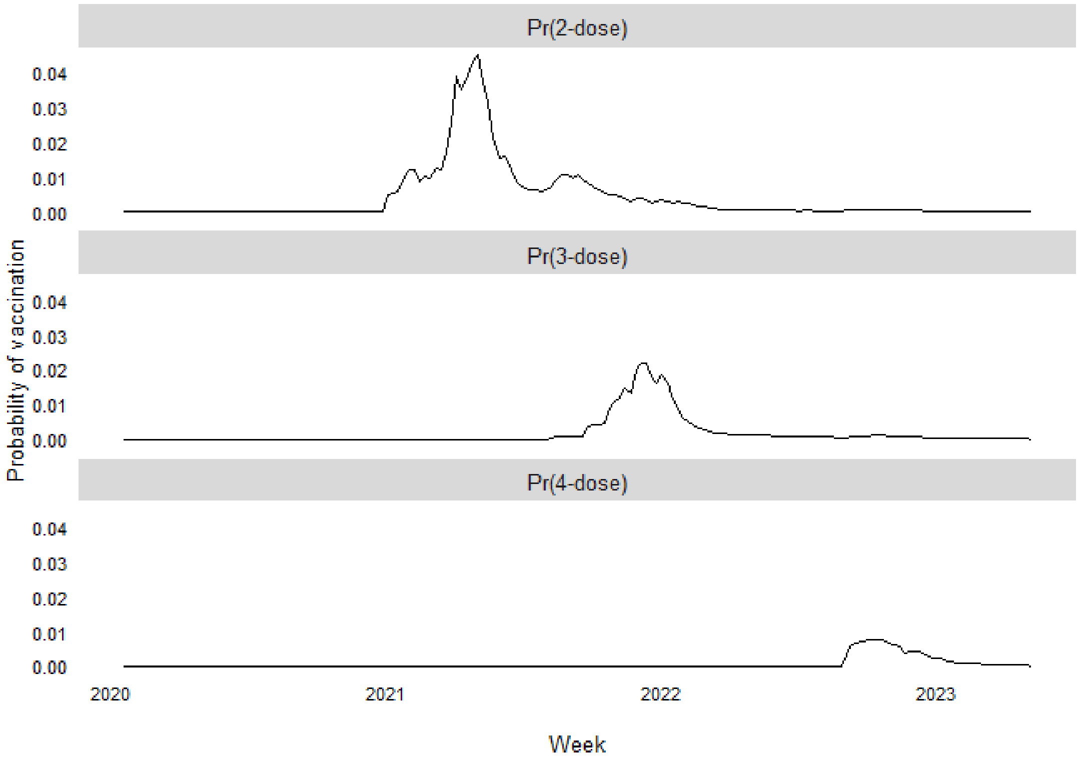

Supplementary Fig. 33: Weekly probabilities of a vaccination dose receipt based on daily vaccination data and U.S. population estimates.

The curves were standardized so that the cumulative probability of vaccination equaled 1 and then fit to a Beta distribution to find an estimated, smoothed curve of vaccination probability by week (Supplementary Fig. 34).

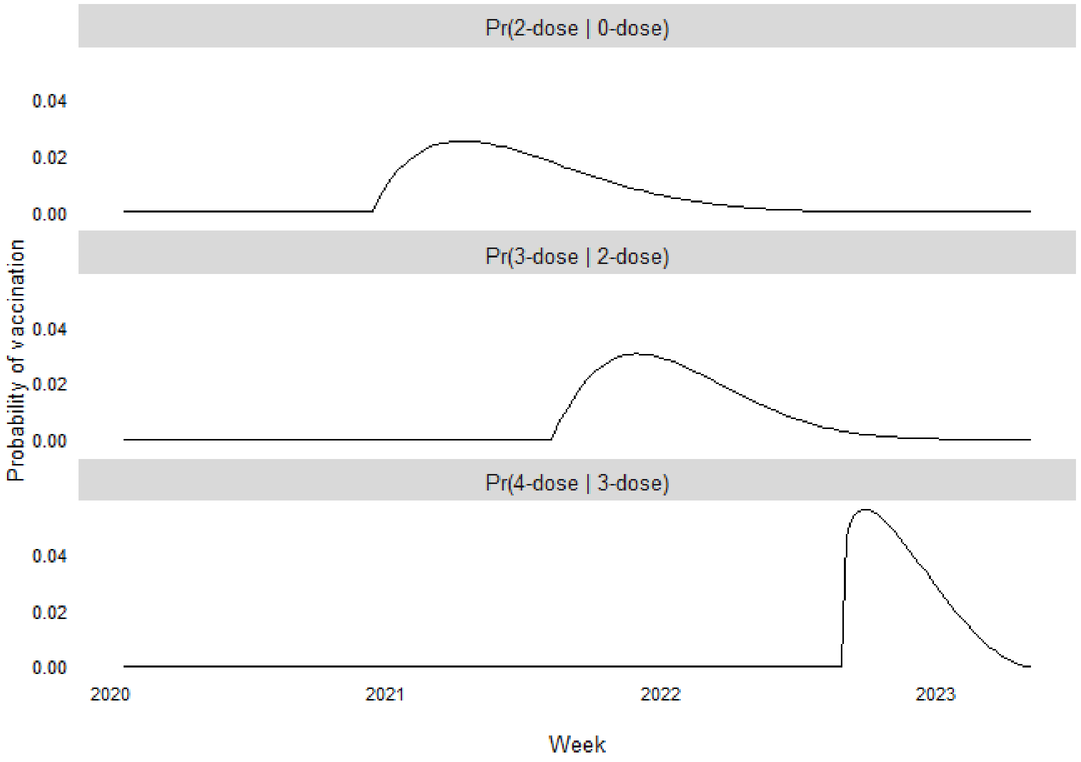

Supplementary Fig. 34: Beta distributions of weekly probability of an additional vaccination dose conditional on a person having had the prior vaccination dose.

In addition, research suggests that people have been less likely to obtain a COVID-19 vaccination after a known SARS-CoV-2 infection. Multiple studies explored whether people with prior infection were less likely to get vaccinated than people without a known prior infection, each with slightly different questions posed to participants. In our search of the literature, we found the following odds ratios (ORs) with the specific comparison estimated:

- Probably/definitely will not get a vaccine: OR=0.63757. [5]
- Reachable vs. reluctant: OR=0.62361. [6]
- Plans to receive vaccination: OR=0.55. [7]
- Intention to receive vaccine: OR=0.40. [8]
- Self-reported past vaccination: OR=0.50. [9]
- Receiving or planning to receive vaccination: OR=0.45833. [10]

Please note our search may not have been exhaustive. From those studies, the median OR was 0.525 which was used in simulations. In our simulations, the ratios did not depend on the dose, e.g., an unvaccinated person was treated the same as someone with three vaccination doses

In an attempt to realize the median OR at each week k, the expected marginal totals were calculated for each combination of $D'_{j,k}$ and $D_{j,k}$. Those marginal totals were naive people ($n_{1,k}$), people with a prior infection ($n_{2,k}$), expected number of people with a vaccination in week k ($n_{3,k}$), and expected number of people without a vaccination in week k ($n_{4,k}$). The marginal totals were used to calculate the probabilities of vaccination in people with prior infection and naive people are determined. That was done by solving the formula

$$\frac{\omega_k * (n_{1,k} - n_{3,k} + \omega_k)}{(n_{3,k} - \omega_k)(n_{2,k} - \omega_k)} - \eta = 0, \tag{1}$$

where $\eta = 0.525$ and ω_k was the number of vaccinations in prior infected people in week k. Then

$$\Pr(D'_{j,k} \mid D_{j,k}, I^*_{j,k}) = \begin{cases} \dfrac{n_{3,k} - \omega_k}{n_{1,k}} & \text{if } I^*_{j,k} = 0, \\ \dfrac{\omega_k}{n_{2,k}} & \text{if } I^*_{j,k} = 1. \end{cases} \tag{2}$$

Protection

Protection in week k was defined based a person's vaccine-induced protection (VP) and infection-induced protection (IP) in week $k - 1$. Each used the same basic function: for VP, the function is as follows:

$$\mathrm{VP}_{j,k} = f_v(k - 1, t^v_{j,k-1}, \kappa_v, \theta_v) = \max\left[0, \kappa_v + \frac{\kappa_v}{-\theta_v} * (k - 1 - t^v_{j,k-1})\right], \tag{3}$$

where $t^v_{j,k-1}$ was the week of the most recent vaccination in week $k - 1$, κ_v was the maximum protection conferred by vaccination, and θ_v was the number of weeks before protection from vaccination reaches zero.

Two definitions were used for vaccination-induced protection waning with different curves for pre-Omicron and Omicron/post-Omicron (Supplementary Fig. 35).

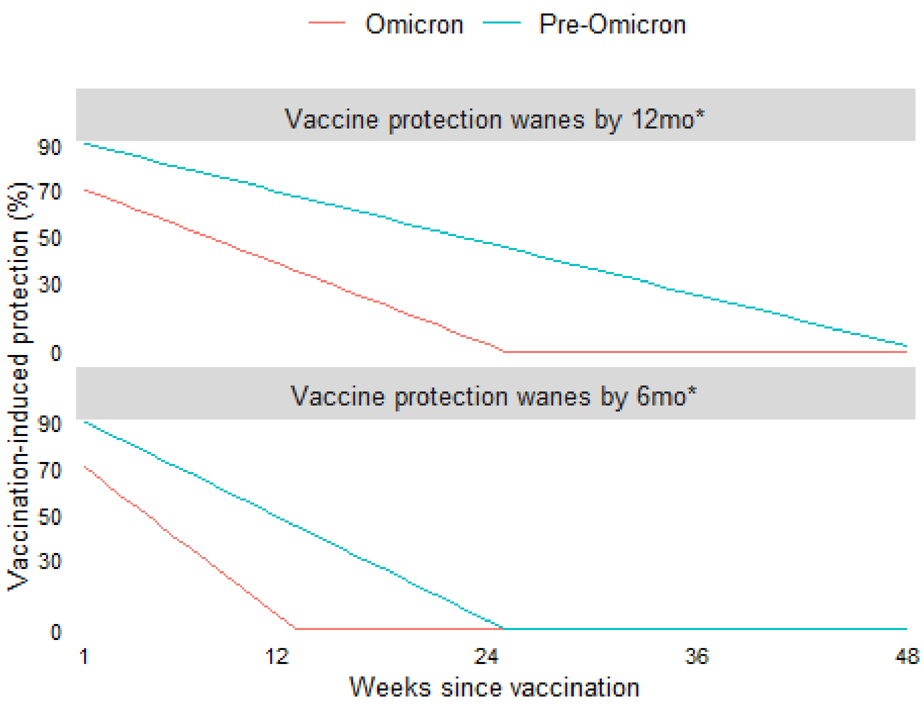

Supplementary Fig. 35: Vaccine protection waning curves.

An individual's vaccination-induced protection level was varied via a multiplier. That multiplier was generated from a Gamma distribution with $\Gamma(\alpha, \beta)$ where α was the shape parameter and β the scale. For this distribution, $\alpha = 100$ and $\beta = 0.01$ (Supplementary Fig. 36).

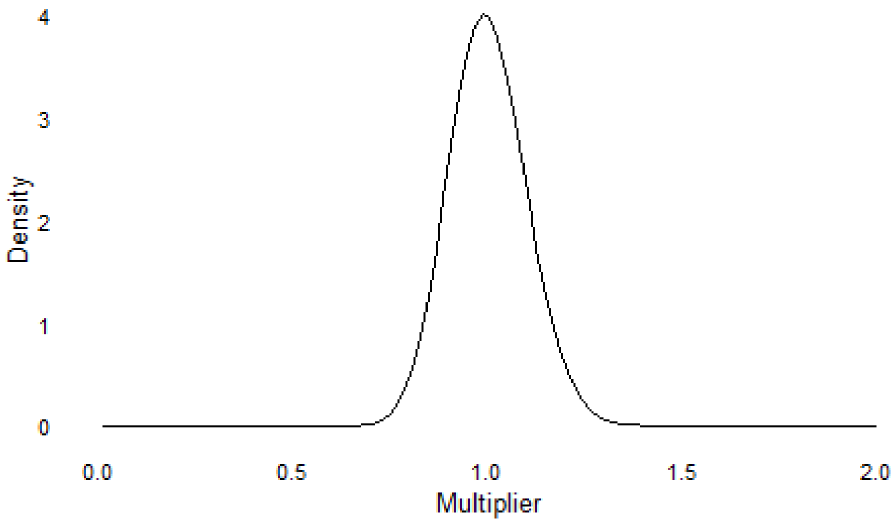

Supplementary Fig. 36: Distribution of vaccine-induced protection multipler.

A similar function to VP was used for IP:

$$\mathrm{IP}_{j,k} = f_p\left(k-1, t_{j,k-1}^{p}, \kappa_p, \theta_p\right) = \max\left[0, \kappa_p + \frac{\kappa_p}{-\theta_p} * \left(k-1-t_{j,k-1}^{p}\right)\right], \quad (4)$$

where $t_{j,k-1}^{p}$ was the week of the most recent infection in week $k-1$, κ_p was the maximum protection conferred by prior infection, and θ_p was the number of weeks before protection from a prior infection reaches zero.

Two definitions were used for infection-induced protection with the same curves regardless of variant (Supplementary Fig. 37).

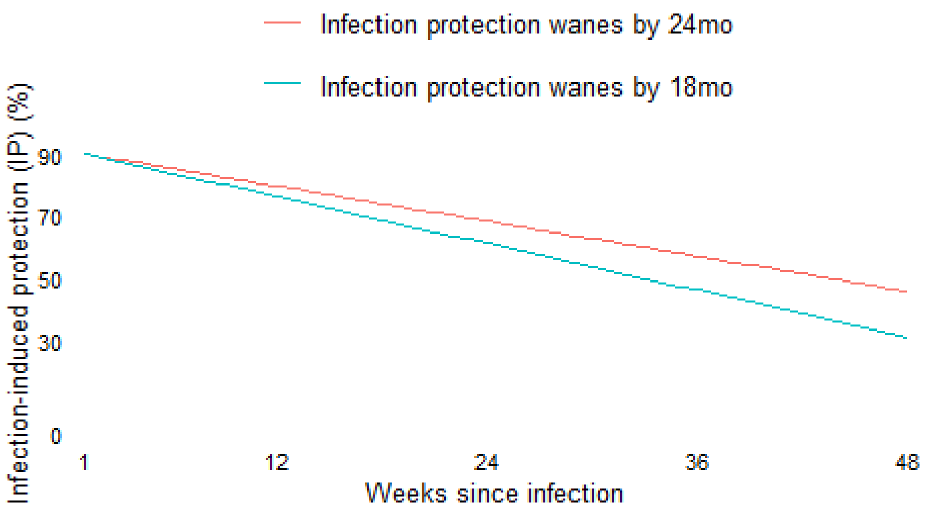

Supplementary Fig. 37: Infection protection waning curves.

As with vaccination-induced protection, we included a multiplier for each individual's infection-induced protection. The distribution was the same as for infection-induced protection, specifically, a $\Gamma(\alpha, \beta)$ with $\alpha = 100$ and $\beta = 0.01$ (Supplementary Fig. 38).

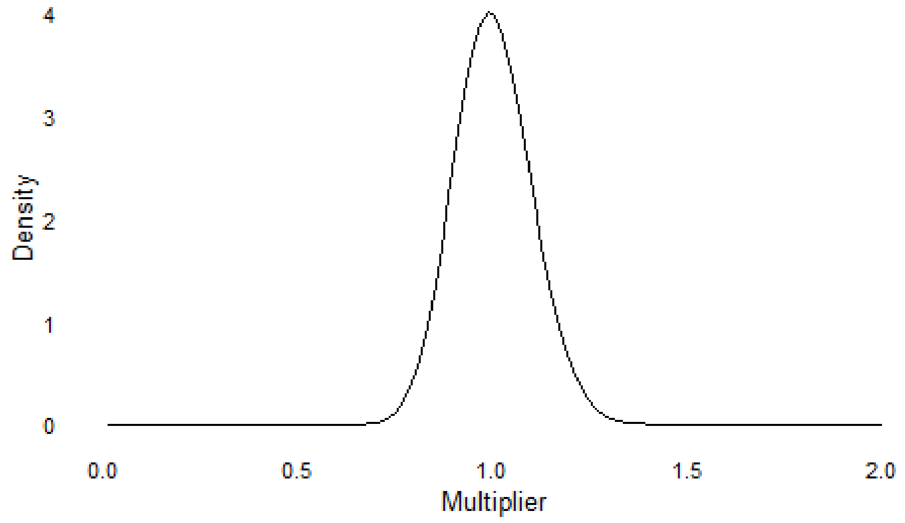

Supplementary Fig. 38: Distribution of infection-induced protection multipler.

For people with both vaccine-induced protection and infection-induced protection, hybrid protection (or hybrid immunity) was determined by one of two definitions and used for $\mathrm{HP}_{j,k}$.

One scenario was to have the larger of the vaccine-induced and infection-induced protection boosted by 30% of the other protection up to a maximum of 0.99 (or 99%) (Supplementary Fig. 39):

$$\mathrm{HP}_{j,k} = \begin{cases} \min\left[0.99,\ \mathrm{IP}_{j,k} + 0.3 * \mathrm{VP}_{j,k}\right] & \text{if } \mathrm{IP}_{j,k} \geq \mathrm{VP}_{j,k} \\ \min\left[0.99,\ \mathrm{VP}_{j,k} + 0.3 * \mathrm{IP}_{j,k}\right] & \text{if } \mathrm{IP}_{j,k} < \mathrm{VP}_{j,k}. \end{cases} \tag{5}$$

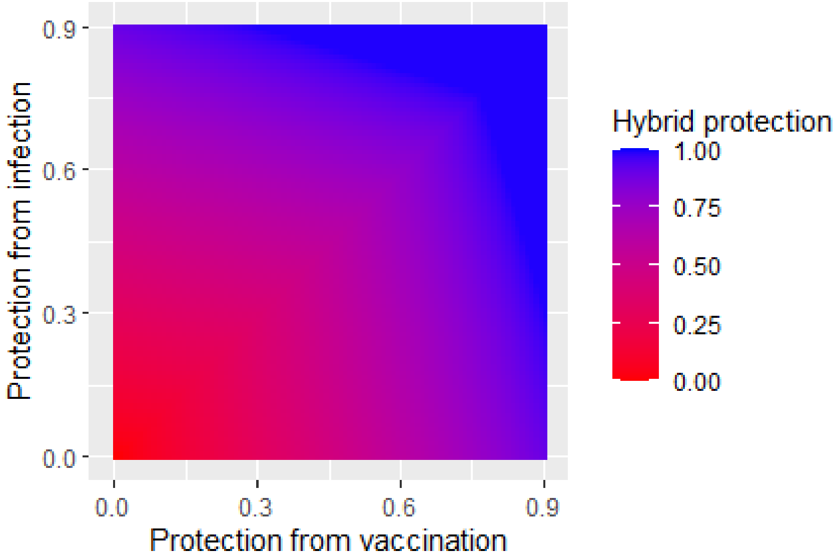

*Supplementary Fig. 39: Hybrid protection that is a maximum of 30% percent boost (either IP + 0.3 * VP or VP + 0.3 * IP).*

The other scenario boosted protection but unequally based on which type of protection was larger, i.e.,

$$\mathrm{HP}_{j,k} = \begin{cases} \min\left[0.99,\ \mathrm{VP}_{j,k} + 0.3 * \mathrm{IP}_{j,k}\right] & \text{if } \mathrm{VP}_{j,k} \geq \mathrm{IP}_{j,k} \\ \min\left[0.99,\ \mathrm{IP}_{j,k} + 0.1 * \mathrm{VP}_{j,k}\right] & \text{if } \mathrm{VP}_{j,k} < \mathrm{IP}_{j,k}. \end{cases} \tag{6}$$

In this scenario, a person's vaccine-induced protection was again boosted by 30% of that person's infection-induced protection up to a maximum of 0.99. On the other hand, infection-induced protection was only boosted by 10% of vaccine-induced protection up to a maximum of 0.99 (Supplementary Fig. 40).

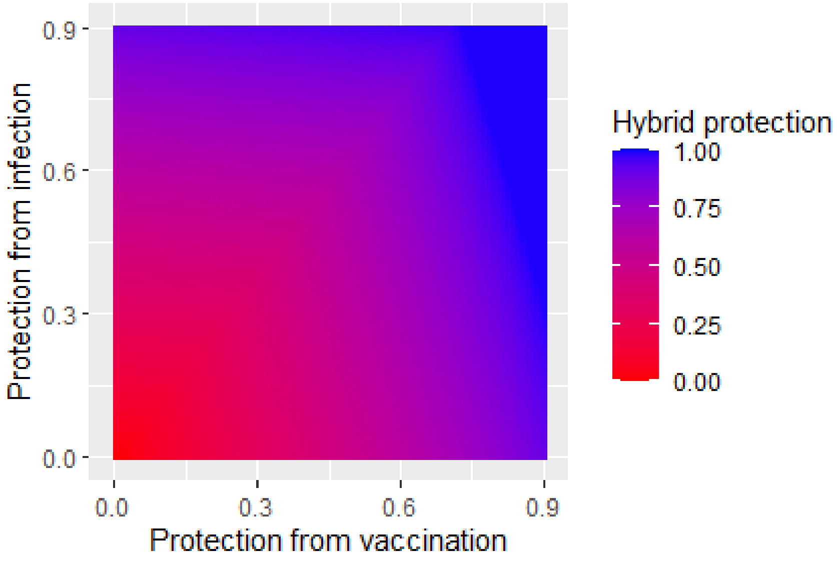

*Supplementary Fig. 40: Hybrid protection that is a maximum of unequal percent boost (either IP + 0.1 * VP or VP + 0.3 * IP).*

Once $\text{HP}_{j,k}$ was determined, person j's protection that week ($\psi_{j,k}$) was

$$\psi_{j,k} = \begin{cases} 0 & \text{if } \text{IP}_{j,k} = 0 \text{ and } \text{VP}_{j,k} = 0 \\ \text{IP}_{j,k} & \text{if } \text{IP}_{j,k} > 0 \text{ and } \text{VP}_{j,k} = 0 \\ \text{VP}_{j,k} & \text{if } \text{IP}_{j,k} = 0 \text{ and } \text{VP}_{j,k} > 0 \\ \text{HP}_{j,k} & \text{if } \text{IP}_{j,k} > 0 \text{ and } \text{VP}_{j,k} > 0. \end{cases} \quad (7)$$

Generation of infection and vaccination

The probability of infection for person j in week k was

$$\Pr(I_{j,k}) = \Pr(c_k) * (1 - \psi_{j,k-1}) \quad (8)$$

and $I_{j,k} \sim Bern\left(\Pr(I_{j,k})\right)$ where $I_{j,k}$ was the infection status for person j in week k where $I_{j,k} = 1$ if infected and $I_{j,k} = 0$ if uninfected.

The additional vaccination dose for person j in week k, denoted by $V_{j,k}$, was $V_{j,k} \sim Bern\left(\Pr(D'_{j,k})\right)$. An additional vaccination dose does not impact protection until the following week, thus

$$D_{j,k+1} = \begin{cases} D_{j,k} & \text{if } V_{j,k} = 0 \\ D'_{j,k} & \text{if } V_{j,k} = 1. \end{cases} \quad (9)$$

Sixteen different parameter sets were used to generate historical data. These consisted of all combinations of two definitions of VP waning, two definitions of IP waning, and four definitions of hybrid protection.

A total of 200 populations were created for each of the four parameter sets.

Example plot of protection

The image below is an example from a simulation of 100 people to show the changing immunity over time prior to the TND (Supplementary Fig. 41).

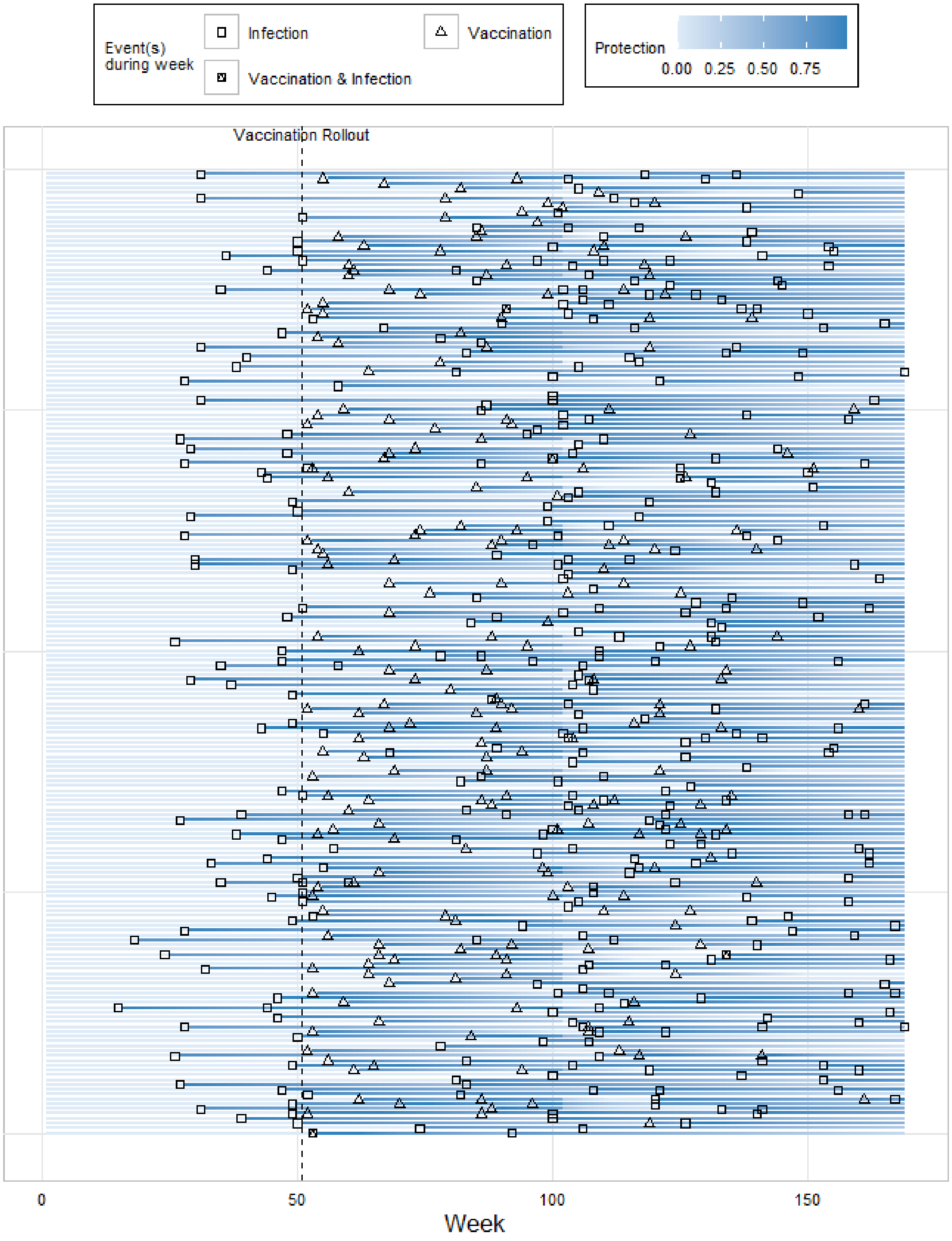

Supplementary Fig. 41: Example of protection trajectories during the historical period for 100 people.

Part 2: analytic period

For the analytic period, eight parameters were varied:

1. two definitions of infection-induced protection;
2. two definitions of vaccination-induced protection;
3. two definitions of hybrid protection;
4. four case distributions presented (constant 2%, constant 4%, 2% with a 4% peak, 2% with a 6% peak);
5. two outcomes (symptomatic infection or severe disease);
6. two vaccination percentages for the population (10%, 25%);
7. three time intervals for the vaccination rollout (weeks 1-12 [before the case peak], weeks 11-22 [during the case peak], or weeks 21-32 [after the case peak]); and
8. three time intervals for the TND against symptomatic infection (weeks 1-12 [before the case peak], weeks 11-22 [during the case peak], or weeks 21-32 [after the case peak]; TND against severe disease only one time interval of weeks 13-32).

The formulas for $\Pr(I_{j,k})$ and $\Pr(D'_{j,k} \mid D_{j,k}, I^*_{j,k})$ remained the same. Simulations were run for each combination for a total of 768 combinations. Each simulated population was used five times for each parameter set.

Probability of cases

Four case distributions were simulated (Supplementary Fig. 42). As before, the probabilities assumed no existing protection.

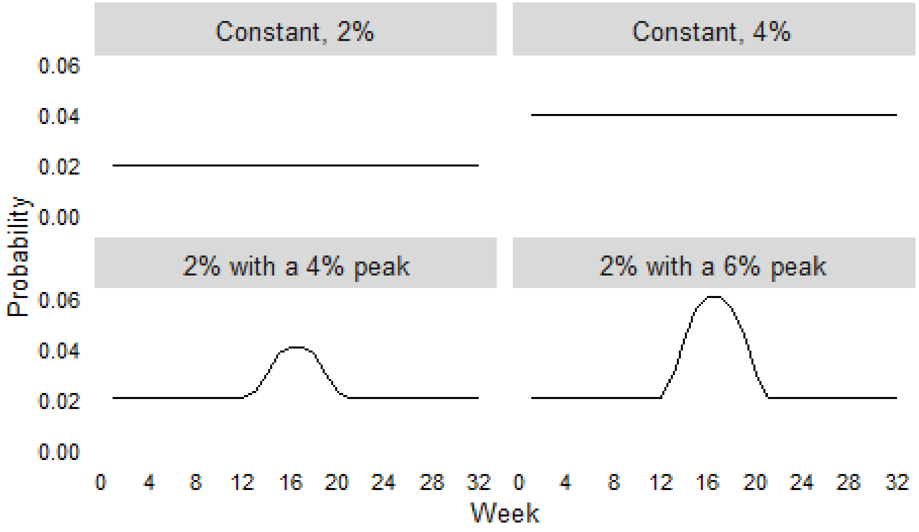

Supplementary Fig. 42: Case distributions during the analytic period.

Probability of vaccination

The vaccination rollout distribution was generated from a lognormal distribution with a mean of 1.5 and a standard deviation of 0.5 and could occur before the mode in the case distribution (weeks 1-12), during the mode in the case distribution (weeks 11-22), or after the mode in the case distribution (weeks 21-32) (Supplementary Fig. 43).

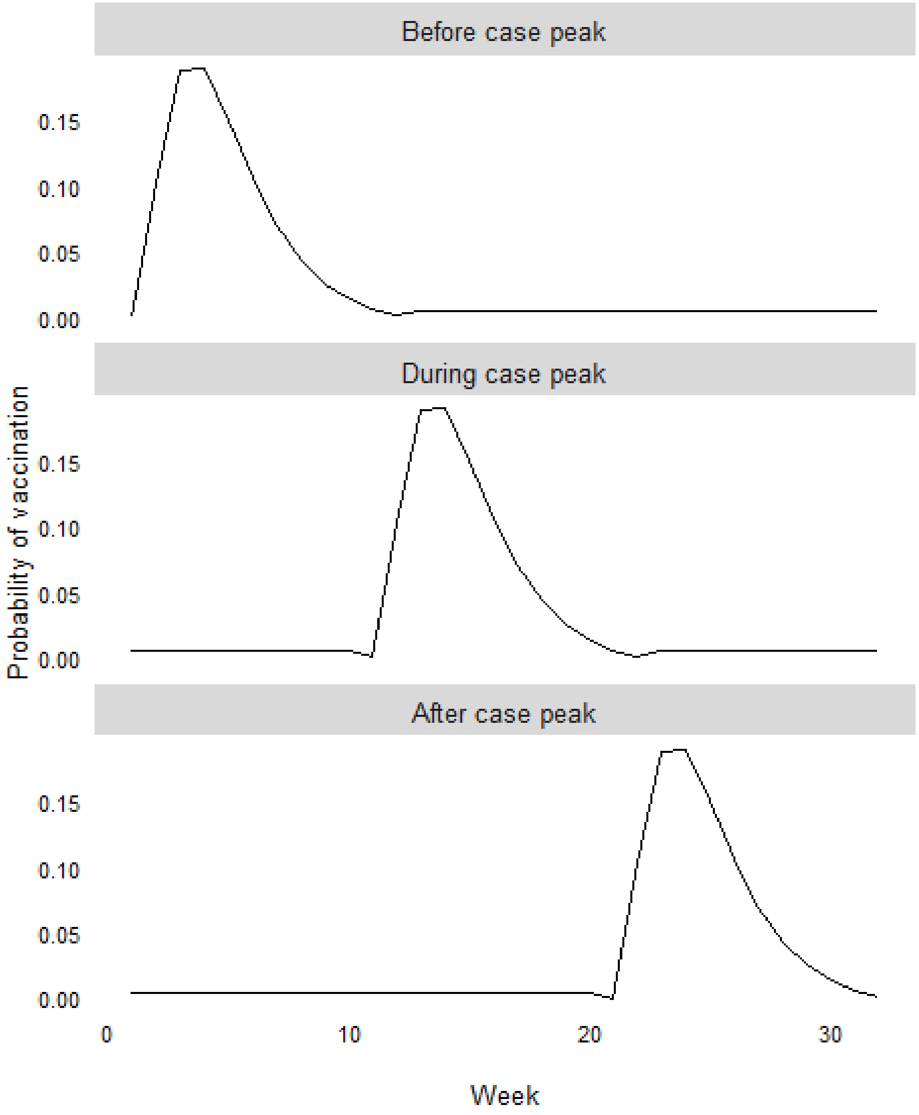

Supplementary Fig. 43: Distributions of vaccination coverage used in simulations.

The total probability of vaccination over the 32-week period was set at 10% or 25% and the above curve was scaled to match (Supplementary Fig. 44).

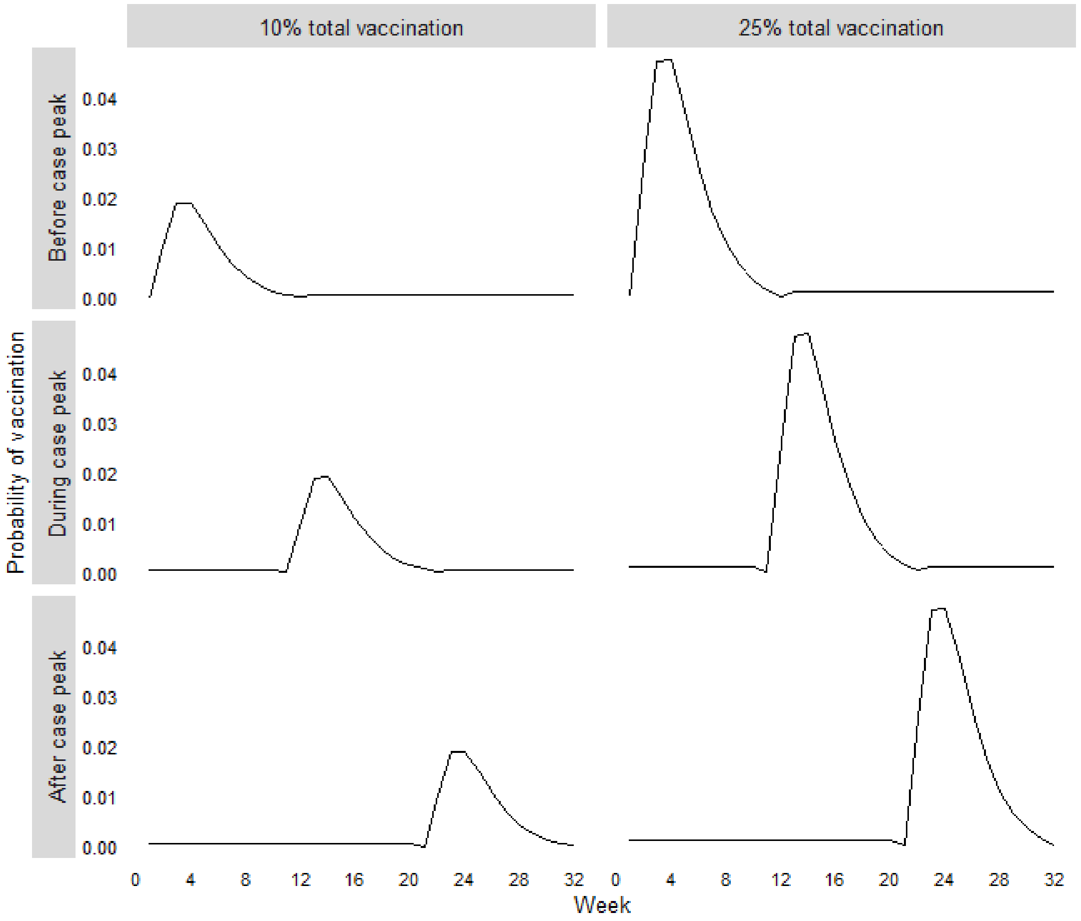

Supplementary Fig. 44: Final weekly distributions of seasonal vaccination coverage used in simulations.

Symptoms

For infected people, the expected probability of symptoms was 0.8 and for uninfected people, the probability of symptoms was 0.2. This parameter was varied by individual to introduce some randomness to an infected person's (Supplementary Fig. 45) or uninfected person's (Supplementary Fig. 46) proclivity to develop symptoms after an infection. Of note, for uninfected people, the likelihood of symptoms is defined as the probability of symptoms over the analytic period instead of the probability of symptoms for an infection event.

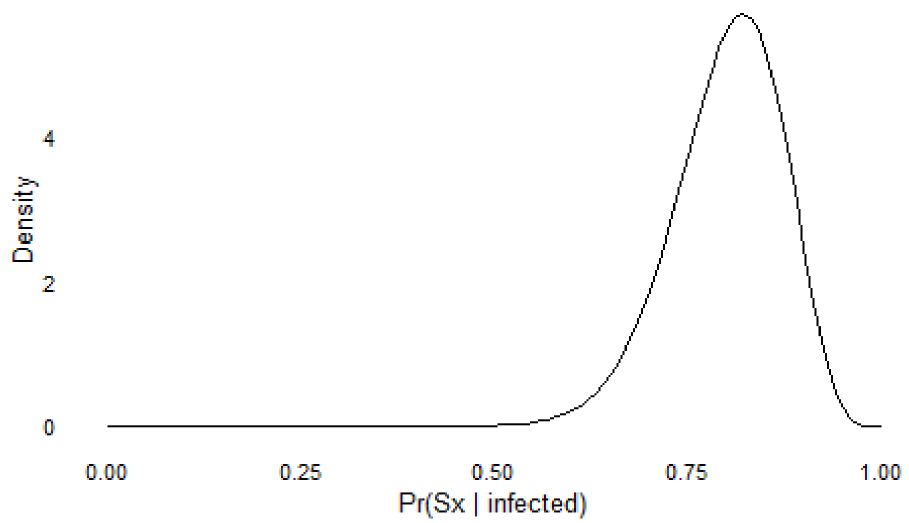

Supplementary Fig. 45: Distribution of the probabilty of symptoms for an infected person during the week of infection.

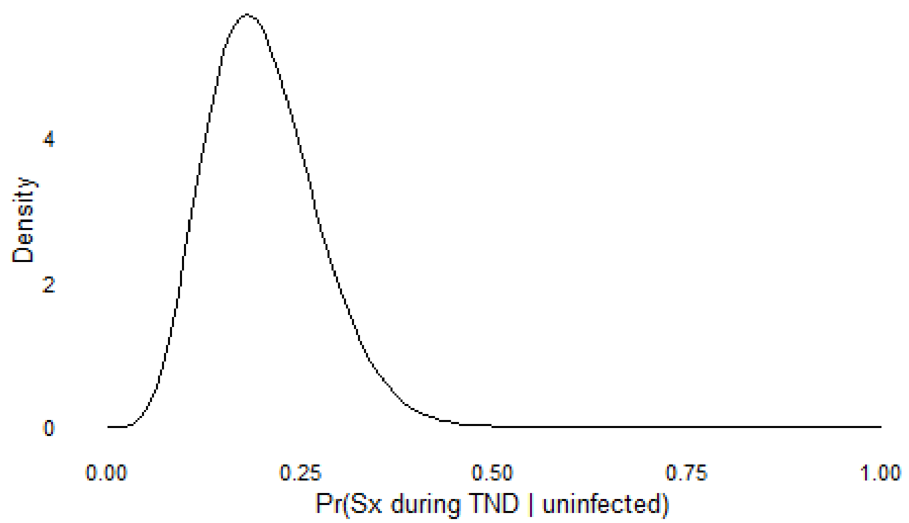

Supplementary Fig. 46: Distribution of the probabilty of symptoms for an uninfected person during the entire analytic period.

Test Characteristics

For the symptomatic infection outcome, testing positive depended on the weeks since the infection. Sensitivity values were dependent on the weeks since infection based on the results in Miller et al. [11]

Supplementary Table 4: Test characteristics by week where week=0 indicates the week of infection.

days	week	Sensitivity	Specificity
0-7	0	90	100
8-14	1	70	100
15-21	2	50	100
22-28	3	20	100
29-35	4	5	100
36+	5+	0	100

Symptomatic infection outcome

For the symptomatic infection outcome, whether a person was symptomatic in the week of data collection was included in the simulations. Once a person was symptomatic, they are automatically included in the TND at that week. Symptomatic people were then tested and, as covered above, the sensitivity of the test depends on how many weeks since a person's last infection. A positive test was randomly generated from a Bernoulli distribution with probability equal to the test sensitivity divided by 100.

Severe disease outcome

Vaccination-induced protection against severe disease started at 0.9 and took 192 weeks to wane to zero. Infection-induced protection against severe disease starts slightly higher at 0.95 and takes longer, specifically 384 weeks, to wane to zero (Supplementary Fig. 47).

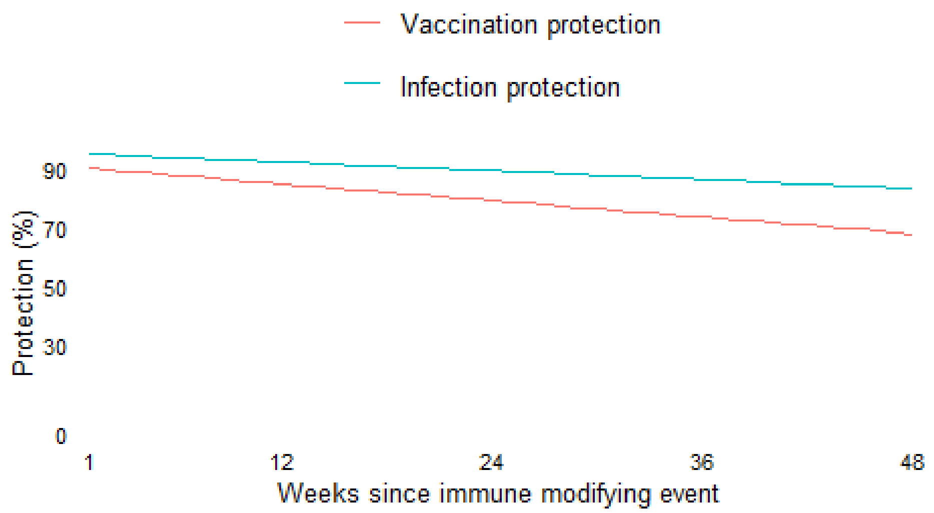

Supplementary Fig. 47: Waning curves for protection against severe disease.

Hybrid protection against severe disease was determined using the same function as hybrid protection against infection.

To generate severe disease, $\Pr(I_{j,k})$ was simulated to create an infection outcome. Those infected in that week ($I_{j,k} = 1$) were determined to have a severe outcome by

$$S_{j,k} \sim Bern\left(\Pr(S_{j,k} \mid I_{j,k} = 1)\right) \tag{10}$$

where

$$\Pr(S_{j,k} \mid I_{j,k} = 1) = \frac{(1 - \psi^{s}_{j,k-1})}{(1 - \psi_{j,k-1})} \tag{11}$$

where $\psi^{s}_{j,k-1}$ was person j's protection against severe disease calculated after week $k - 1$.

For uninfected people, i.e., $I_{j,k} = 0$, we set

$$\Pr(S_{j,k} \mid I_{j,k} = 0) = \frac{0.02}{20}, \tag{12}$$

where $S_{j,k}$ was a severe disease event, 0.02 was chosen so the expectation would be that 2% of people would experience a severe disease event, and 20 is the number of weeks in the severe disease TND.

Testing was considered perfect (sensitivity=specificity=1) as we assumed a patient with severe respiratory disease would be diagnosed correctly.

Analytic methods

Models

Exposures fit in the models were:

1. vaccination at any time during the analytic period;
2. vaccination in the previous 2 months;
3. vaccination in the previous 3 months;
4. vaccination in the previous 4 months;
5. vaccination in the previous 5 months;
6. vaccination in the previous 6 months;
7. the number of doses received (unvaccinated as the reference group, 2-dose, 3-dose, 4-dose, or 5-dose); and
8. the time since vaccination (unvaccinated as the reference group, 0-2 months, 3-4 months, 5-11 months, and 12 or more months).

Each exposure was included in a model with either

1. no other covariates (uncontrolled or unadjusted model) or
2. the months since the last infection (categorical with months = $\{1,2,\ldots,11,12 \text{ or more}\}$) and the number of prior infections as a continuous variable (controlled or adjusted model).

For each logistic regression, we modeled $\Pr\left(I^*_{j,k}\right)$, such that

$$\Pr\left(I^*_{j,k}\right) = \frac{\exp(\zeta)}{1+\exp(\zeta)}. \tag{13}$$

Corresponding to each of the exposure definitions, the unadjusted models were parameterized as follows:

1. $\zeta = \beta_0 + \beta_1 \mathrm{AP}_{j,k}$, where $\mathrm{AP}_{j,k} = 1$ if person j got a vaccination in the simulated roll out by week k and $\mathrm{AP}_{j,k} = 0$ if not;
2. $\zeta = \beta_0 + \beta_1 \mathrm{R}^{2M}_{j,k}$, where $\mathrm{R}^{2M}_{j,k} = 1$ if person j got a vaccination in the 2 months prior to week k and $\mathrm{R}^{2M}_{j,k} = 0$ if not;
3. $\zeta = \beta_0 + \beta_1 \mathrm{R}^{3M}_{j,k}$, where $\mathrm{R}^{3M}_{j,k} = 1$ if person j got a vaccination in the 3 months prior to week k and $\mathrm{R}^{3M}_{j,k} = 0$ if not;
4. $\zeta = \beta_0 + \beta_1 \mathrm{R}^{4M}_{j,k}$, where $\mathrm{R}^{4M}_{j,k} = 1$ if person j got a vaccination in the 4 months prior to week k and $\mathrm{R}^{4M}_{j,k} = 0$ if not;
5. $\zeta = \beta_0 + \beta_1 \mathrm{R}^{5M}_{j,k}$, where $\mathrm{R}^{5M}_{j,k} = 1$ if person j got a vaccination in the 5 months prior to week k and $\mathrm{R}^{5M}_{j,k} = 0$ if not;
6. $\zeta = \beta_0 + \beta_1 \mathrm{R}^{6M}_{j,k}$, where $\mathrm{R}^{6M}_{j,k} = 1$ if person j got a vaccination in the 6 months prior to week k and $\mathrm{R}^{6M}_{j,k} = 0$ if not;

7. $\zeta = \beta_0 + \beta_1 \mathrm{R}_{j,k}^{\text{2-dose}} + \beta_2 \mathrm{R}_{j,k}^{\text{3-dose}} + \beta_3 \mathrm{R}_{j,k}^{\text{4-dose}} + \beta_4 \mathrm{R}_{j,k}^{\text{5-dose}}$, where $\mathrm{R}_{j,k}^{n\text{-dose}} = 1$ if person j had n doses in week k and $\mathrm{R}_{j,k}^{n\text{-dose}} = 0$ if not; and
8. $\zeta = \beta_0 + \beta_1 \mathrm{R}_{j,k}^{0-2\text{ months}} + \beta_2 \mathrm{R}_{j,k}^{3-4\text{ months}} + \beta_3 \mathrm{R}_{j,k}^{5-11\text{ months}} + \beta_4 \mathrm{R}_{j,k}^{12+\text{ months}}$, where $\mathrm{R}_{j,k}^{n\text{ months}} = 1$ if person j had her or his most recent vaccination n months in week k and $\mathrm{R}_{j,k}^{n\text{ months}} = 0$ if not.

In all models, VE was calculated as $(1 - OR) * 100$ where OR was the odds ratio from the logistic model.

Bias

Bias in simulation studies is typically defined as the deviation from truth. In these simulations, true VE is challenging to determine since true VE depends the distribution of vaccination dissemination, the waning protection of vaccinations, the distribution of infections, and the waning protection of infections. Thus, a true VE will depend on each individual's time since vaccination and, if applicable, time since last infection, meaning the true VE will be different for each simulation.

Though, the purpose of these simulations is to present a real-world, policy-relevant evaluation of ignoring the effect of prior infection in an evaluation of vaccine effectiveness. To do this, we felt it was important to generate individual people's histories across a relevant range of scenarios. As a result, these simulations differ from typical simulation studies because we do not compare our results to a true parameter value. Thus, our definition of bias is

$$\mathrm{Bias(VE)} = \mathrm{E}\left[\mathrm{VE}^{\text{unadjusted}} - \mathrm{VE}^{\text{adjusted}}\right] \qquad (14)$$

where $\mathrm{VE}^{\text{unadjusted}}$ was the vaccine effectiveness estimate from models without controlling for participants' prior infection status and $\mathrm{VE}^{\text{adjusted}}$ was the vaccine effectiveness estimate from models that adjust for participants' prior infection.

We retain the use of "bias" to describe our comparison. Although we do not compare our simulated VE estimates to a true VE, we feel bias is a reasonable term to use since that conveys the deviation from an established measurement.

Unstable estimates

A total of 21,504,000 VE estimates were produced from these simulations.

Of those, 260 estimates had a log odds above 1 (corresponding to an odds ratio of 2.72 or greater). Two of those were from the 5-11 months since vaccination category and the other 258 were from the 5-dose exposure group. The odds ratios were large in these simulations since a very small number of people were sampled (median=11; range=4, 23) and a high

percentage were positive. For these simulations, an odds ratio of 1.00 was used, which corresponds to a VE of $(1 - e^1) * 100 = -171.83$.

A total of 4,178 estimates had a large standard error (defined as a standard error of 2 or greater). Two of these estimates were from the vaccination at any time during the analytic period exposure group, 26 were from the 5-11 months since vaccination category, and the remaining 4,150 were from the 5-dose exposure group. These simulations usually had a large standard error because no exposed people were positive for SARS-CoV-2. For these simulations with a large sample size, a standard error of five was used.

Two simulations fell into both categories, meaning a total of 4,436 estimates (or 0.02%) were unstable.

References

1. Centers for Disease Control and Prevention COVID-19 Response. Weekly united states COVID-19 cases and deaths by state. 2023; Available at: \url{https://data.cdc.gov/Case-Surveillance/Weekly-United-States-COVID-19-Cases-and-Deaths-by-/pwn4-m3yp}.

2. Wiegand RE, Deng Y, Deng X, et al. Estimated SARS-CoV-2 antibody seroprevalence trends and relationship to reported case prevalence from a repeated, cross-sectional study in the 50 states and the District of Columbia, United States—October 25, 2020–February 26, 2022. The Lancet Regional Health–Americas **2023**; 18:100403.

3. Centers for Disease Control and Prevention. 2022 nationwide COVID-19 infection- and vaccination-induced antibody seroprevalence (blood donations). 2023; Available at: \url{https://covid.cdc.gov/covid-data-tracker/#nationwide-blood-donor-seroprevalence-2022}.

4. Centers for Disease Control and Prevention COVID-19 Response. COVID-19 vaccination age and sex trends in the united states, national and jurisdictional. 2023; Available at: \url{https://data.cdc.gov/Vaccinations/COVID-19-Vaccination-Age-and-Sex-Trends-in-the-Uni/5i5k-6cmh}.

5. Nguyen KH, Huang J, Mansfield K, Corlin L, Allen JD. COVID-19 vaccination coverage, behaviors, and intentions among adults with previous diagnosis, united states. Emerging Infectious Diseases **2022**; 28:631.

6. Lutrick K, Groom H, Fowlkes AL, et al. COVID-19 vaccine perceptions and uptake in a national prospective cohort of essential workers. Vaccine **2022**; 40:494–502. Available at: https://www.sciencedirect.com/science/article/pii/S0264410X21015930.

7. Pacella-LaBarbara ML, Park YL, Patterson PD, et al. COVID-19 vaccine uptake and intent among emergency healthcare workers: A cross-sectional survey. Journal of Occupational and Environmental Medicine **2021**; 63:852–856.

8. Moniz MH, Townsel C, Wagner AL, et al. COVID-19 vaccine acceptance among healthcare workers in a united states medical center. medRxiv **2021**; Available at: https://www.medrxiv.org/content/early/2021/04/30/2021.04.29.21256186.

9. Do DP, Frank R. Prior COVID-19 infection: an underappreciated factor in vaccine hesitancy in the USA. Journal of Public Health **2022**; 44:471–474. Available at: https://doi.org/10.1093/pubmed/fdab404.

10. Nguyen V-TT, Huang Y, Huang M, Tsai J. Factors related to COVID-19 vaccine hesitancy among middle-income and low-income adults in the USA. Journal of Epidemiology & Community Health **2023**; 77:328–335. Available at: https://jech.bmj.com/content/77/5/328.

11. Miller TE, Garcia Beltran WF, Bard AZ, et al. Clinical sensitivity and interpretation of PCR and serological COVID-19 diagnostics for patients presenting to the hospital. The

FASEB Journal **2020**; 34:13877–13884. Available at: https://faseb.onlinelibrary.wiley.com/doi/abs/10.1096/fj.202001700RR.
