# Supplementary material for: Bias and negative values of COVID-19 vaccine effectiveness estimates from a test-negative design without controlling for prior SARS-CoV-2 infection
Source: Nat Commun. 2024 Nov 20;15:10062. doi: 10.1038/s41467-024-54404-w (PMC11579392; doi:10.1038/s41467-024-54404-w)
Supplement: Supplementary file 2 — Description of Additional Supplementary Files [file 41467_2024_54404_MOESM2_ESM.pdf]

Supplementary Code 1: Code and README file for reproducing analyses in the manuscript.
