# Supplementary material for: Bias and negative values of COVID-19 vaccine effectiveness estimates from a test-negative design without controlling for prior SARS-CoV-2 infection
Source: Nat Commun. 2024 Nov 20;15:10062. doi: 10.1038/s41467-024-54404-w (PMC11579392; doi:10.1038/s41467-024-54404-w)
Supplement: Supplementary file 3 — Supplementary Code 1 [file 41467_2024_54404_MOESM3_ESM.zip › README_NCOMMS-24-45954.pdf]

Code archive README file for 'Bias and negative values of vaccine effectiveness estimates from a test-negative design without controlling for prior infection'

NCOMMS-24-45954

Ryan E. Wiegand, Bruce Fireman, Morgan Najdowski, Mark W. Tenforde, Ruth Link-Gelles, Jill M. Ferdinands

System requirements: R version >= 4 and compatible RStudio version.
Operating system used: Windows 10 Enterprise
R versions used: 4.0.4 and 4.4.0

Installation guide

1. Install R from https://cran.r-project.org/.
2. Install RStudio from https://posit.co/download/rstudio-desktop/.
3. It is also advised to download Rtools from https://cran.r-project.org/.

Installation time for a normal computer is unknown but expected to be less than 10 minutes with reliable and fast internet connection.

R packages needed for analyses are included in individual scripts.

PLEASE NOTE:

1. There is no dataset associated with these scripts. All data used was simulated and can be created via scripts.
2. These scripts were run in parallel on a high performance cluster (HPC). It is possible to run these scripts without an HPC, but it will take along time.
3. If any scripts are missing, please contact fwk2@cdc.gov.

Description of simulation scripts

- **fig.i.v2.R**: R script with code to create plots for vaccine effectiveness (VE) against symptomatic infection.
- **fig.s.R**: R script with code to create plots for VE against severe disease.
- **fig.v2.R**: R script with code to create plots for either VE against symptomatic infection or severe disease.
- **metareg_fcn_sensitivity_v1.R**: R script with functions used in tnd_reinfection_metareg_sensitivity.R.
- **metareg_fcn_v4.R**: R script with functions used in tnd_reinfection_metareg.R.
- **non_tnd_loop_v4.R**: R script with a for loop that is used to generate weekly infection and vaccination data for time periods without a test negative design.
- **pop_tnd_reinfection.sh**: Shell script that runs tnd_reinfection_pop_sim_hpc.R on a high performance cluster.
- **tnd_loop_sev_v8.R**: R script with a for loop that is used to generate weekly infection and vaccination data for time periods with a test negative design evaluating vaccine effectiveness against severe disease.

- **tnd_loop_v6.R**: R script with a for loop that is used to generate weekly infection and vaccination data for time periods with a test negative design evaluating vaccine effectiveness against infection.
- **tnd_prior_infection_supplement_unedited.Rmd**: R markdown script that creates manuscript output and an unedited version of the supplementary materials (table of contents, running head, and page numbers are added in Microsoft Word).
- **tnd_reinfection.sh**: Shell script that runs tnd_reinfection_sim_hpc.R on a high performance cluster.
- **tnd_reinfection_metareg.R**: R script that implements meta-regressions to summarize simulation results.
- **tnd_reinfection_metareg_sensitivity.R**: R script that implements meta-regressions to summarize simulation results.
- **tnd_reinfection_param.R**: R script that creates an .RData file with all simulation parameters.
- **tnd_reinfection_pop_sim_hpc.R**: R script that simulates 200 populations for each of 8 historical parameter sets.
- **tnd_reinfection_pop_sum_sim_hpc.R**: R script that creates summaries of the 200 populations for each set of historical period parameters.
- **tnd_reinfection_popsum.sh**: Shell script that runs tnd_reinfection_pop_sum_sim_hpc.R on a high performance cluster.
- **tnd_reinfection_sim_hpc.R**: R script that performs 1,000 simulations from the analytic period for each set of analytic period parameters.
- **tnd_reinfection_summary.R**: R script that creates summaries of 1,000 simulations from analytic period for each set of analytic period parameters.

R and R markdown scripts should be run in the following order:

1. tnd_reinfection_param.R
2. pop_tnd_reinfection.sh (which implements tnd_reinfection_pop_sim_hpc.R)
3. tnd_reinfection.sh (which implements tnd_reinfection_sim_hpc.R)
4. tnd_reinfection_popsum.sh (which implements tnd_reinfection_pop_sum_sim_hpc.R)
5. tnd_reinfection_summary.R
6. tnd_reinfection_metareg.R
7. tnd_reinfection_metareg_sensivitity.R
8. tnd_prior_infection_supplement_unedited.Rmd

Expected runtimes:

1. tnd_reinfection_param.R: under 5 minutes.
2. tnd_reinfection_pop_sim_hpc.R: 7-9 hours.
3. tnd_reinfection_sim_hpc.R: varies by parameter set. Expected to be between 4 and 16 hours per parameter set.
4. tnd_reinfection_pop_sum_sim_hpc.R: 15 minutes for each parameter set.
5. tnd_reinfection_summary.R: under 5 minutes.
6. tnd_reinfection_metareg.R: 15 minutes.
7. tnd_reinfection_metareg_summary.R: 15 minutes.
8. tnd_prior_infection_supplement_unedited.Rmd: under 5 minutes.
